# Mediators of physical activity behaviour change among adult non-clinical populations: a review update

**DOI:** 10.1186/1479-5868-7-37

**Published:** 2010-05-11

**Authors:** Ryan E Rhodes, Leila A Pfaeffli

**Affiliations:** 1 Behavioural Medicine Laboratory, Faculty of Education, University of Victoria, Victoria, BC, Canada; 2 School of Exercise Science, Physical and Health Education, University of Victoria, Victoria, BC, Canada

## Abstract

**Background:**

An understanding of the determinants of physical activity through mediators of behaviour change is important in order to evaluate the efficacy of interventions. Prior reviews on this topic noted that few studies employed mediator analyses in experimental physical activity trials; the purpose of this review is to update these prior reviews in order to evaluate the state of our present understanding of interventions that include proposed mediators of behaviour change.

**Methods:**

Literature was identified through electronic database (e.g., MEDLINE, psychINFO) searching. Studies were eligible if they described a published experimental or quasi-experimental trial examining the effect of an intervention on physical activity behaviour and mediator change in non-clinical adult populations. Quality of included studies was assessed and the analyses examined the symmetry between mediators and behaviour change.

**Results:**

Twenty seven unique trials passed the eligibility criteria and 22 were included in the analysis with scores of moderate or higher quality. Half of the studies reviewed failed to show an intervention effect on PA. The remaining studies showed evidence that the intervention affected changes in the proposed mediators, but tests of mediated effect were performed in only six of these 11 cases and demonstrated mixed outcomes. Differences by theory were not discernable at this time, but self-regulation constructs had the most evidence for mediation.

**Conclusion:**

Published literature employing mediators of change analyses in experimental designs is still relatively elusive since the time of prior reviews; however, the general null findings of changes in mediating constructs from these interventions are a more timely concern. Changes in self-regulation constructs may have the most effect on changes in PA while self-efficacy and outcome expectation type constructs have negligible but limited findings. Innovation and increased fidelity of interventions is needed and should be a priority for future research.

## Introduction

The health benefits of regular physical activity (PA) are well-established and convincing [1], yet at least half of the populace fail to meet national recommended guidelines [2]. As a result, the promotion of PA is of great importance to public health. Intervention efforts have met with very modest success in changing PA [3,4]. For example, a meta-analysis of PA intervention studies conducted by Hillsdon et al., reports an overall change in behaviour of .31 SD, an effect size that is considered very small by generally accepted behavioural standards [5]. Further, the authors showed that interventions had weak evidence in their capability to make behavioural changes at recommended guideline values. Thus, there is a need to hone existing interventions and to make effective and innovative changes.

At the forefront of these considerations is the application of sound behavioural theory when designing interventions [6]. There has been a proliferation of correlation-based theory testing in the general health behaviour domain with recent advocacy for experimental testing [7,8]. Although such tests are undoubtedly essential to establishing the internal validity of a theory, they also have important and immediate applied value to public health promotion efforts. That is, the constructs used in behavioural theories can help us understand "why" or "why not" a PA intervention worked [3]. This seems essential information in the designing of interventions; those PA promotion initiatives constructed to change important target variables should then lead to desired behaviour change, while those interventions used to target ineffective variables can be discarded.

The heart of this argumentation is the assumption of a mediating framework between theoretical constructs and behaviour [3,9]. The assumption in behavioural theory is that interventions can target change in critical antecedents of behavioural engagement and these will follow a causal chain to ensuing behaviour change. Specifically, mediation is achieved with evidence of a significant and substantive product-of-coefficient estimate where the independent variable (e.g., intervention) has its effect on the outcome (e.g., change in PA) via the mediator [10,11]. Currently, behavioural theories/models such as social cognitive theory (SCT) [12], transtheoretical model (TTM) [13], self-determination theory (SDT) [14], and theory of planned behaviour (TPB) [15] are the dominant frameworks for mediating constructs in the PA domain.

Reviews by Baranowski et al. [3] and Lewis et al. [9], have focused on the evaluation of the mediating model in PA interventions. Overall, Baranowski and colleagues noted several limits to the extant literature which were subsequently mirrored in the 10 studies reviewed by Lewis et al. Most strident was the finding that very limited literature had tested the proposed mediating mechanisms with a formal statistical test such as those outlined in Baron and Kenny [16] and more recently the product-of coefficient tests recommended by MacKinnon and colleagues [10]. This information is considered essential for convincing evidence of the causal chain between intervention, theory, and behaviour change. Thus both groups of authors concluded that more research employing formal mediating analyses need to be conducted. Lewis et al. noted in their evaluation of SCT and the TTM that the behavioural processes of change (i.e., self-regulatory actions such as planning, using reinforcements, and cues, etc.) had the most convincing and reliable evidence as a mediator from interventions, but noted that the evidence was still limited. Several tests of interventions and mediators showed mixed or even null relationships with the intervention and PA behaviour in these reviews. Further, no examination of other leading theories such as SDT or the TPB was conducted in their review.

Thus, the purpose of this review was to provide an update of the literature on behavioural mediators of PA interventions since the time of these prior reviews and include all resulting theories applied to PA. The review is also focused on PA as a form of primary prevention among adults so only non-clinical populations were considered. The strong recommendations for formal mediation analyses from these prior reviews coupled with now a seven-year lag in time from the content of Lewis et al. [9] supports the need for a review update.

## Method

### Eligibility criteria

Eligible studies were published journal articles describing an experimental or quasi-experimental trial examining the effect of the theoretical intervention on physical activity behaviour change and on proposed mediating variables. Studies that investigated the relationship between the theoretical variables and the primary outcome of PA were also included. A study was excluded if it examined child, adolescent, older adult (age 65+), or clinical populations. Excluded studies were also those that (1) examined adherence to PA behaviour or stage of change only, (2) did not measure a change in mediating variables, (3) described only the process of the study without stating results, (4) used non experimental designs, or (5) were written in any language other than English (see Additional file 1).

### Search strategy

Literature searchers were conducted from January, 1998 to September, 2008 in ISI Web of Knowledge, SPORTDiscus, psychINFO, and MEDLINE (see Additional file 2). The electronic search strategy was developed by both authors and was based on Baranowski et al. [3] and Lewis et al.'s [9] previous studies examining mediating variables in physical activity interventions. A combination of keywords were used, including physical activity, exercise, physical fitness, psychological theory, psychosocial correlates, intervention, social cognitive theory, transtheoretical model, theory of planned behaviour, self-determination theory, protection motivation theory, behavioural research, theoretical effectiveness, behaviour change, health behaviour, mediator, self-efficacy, cognitive, stage of change, and process of change. The search was executed by one author (LP). The search was not restricted by language, study design, or population. Manual cross-referencing of bibliographies was also completed.

### Screening

Citations were screened by two reviewers (LP, RR) using pre-defined inclusion criteria. Studies were initially screened based on the title and abstract. Relevant abstracts were then selected for a full read of the article. Potential studies for adjudication were examined by two reviewers (RR and LP). It was then determined whether the study met the criteria and was included in the review. Consensus was reached in 100% of the cases.

### Data abstraction

The two authors abstracted data using a pre-specified 12 item data abstraction form (see Additional files 3 and 4). The abstracted data included authors, sample, study design and setting, PA target, dependent variables, intervention theory, intervention length and characteristics, measurement tools, outcomes, and mediator analysis.

### Analysis methods

Studies were grouped in total and by SCT [17], the TTM [13], TPB [18], protection motivation theory (PMT) [19], and SDT [14] based on *a priori *classification of psychological theories [9]. A more specific grouping was also conducted at the construct level across theories. Some prominent theorists have suggested that popular theories of health behaviour have considerable conceptual overlap among their constructs [6,12,18,20]. Using these taxonomies as a guide, constructs of self-efficacy/control (i.e., self-efficacy, perceived behavioural control), outcome expectations (outcome expectations, attitude/behavioural beliefs, pros, cons, response efficacy, vulnerability, severity), self-regulatory processes or goals (intention, planning, goals, self-regulation, behavioural processes) and social expectancies (social support, subjective norm) were included.

Study quality was assessed using the checklist tool developed specifically for mediator analyses by Lubans, Foster and Biddle [21] and three additional items (i.e., measure reliability, appropriate analysis methods, assessment of change in mediator preceding change in the outcome) from Cerin and colleagues [22]. The tool was created with similar scoring to the Cochrane Collaboration's instrument for assessing risk of bias [23] and includes 11 questions answered with a yes (1) or no (0) format. High quality is considered with scores of nine to 11, moderate quality was considered with scores of five to eight and low quality was considered with scores of zero to four. Following the suggestions of the Grade Working Group [24], the overall quality of the studies was reported to describe the general state of research on the topic and this included low quality studies. This was followed by analyses of the high and moderate quality studies, however, in order to make judgements with some protection from risk of bias [21,23].

Studies were coded by whether the intervention was effective in changing behaviour and subsequently with an approach used by Cerin and colleagues [22] that outlines mediator models by tests of action theory, conceptual theory, and simultaneous test of both action and conceptual theories (i.e., mediated effect). Specifically, the action theory test examines whether the intervention was able to change the proposed mediator; the conceptual theory test examines whether intervention-induced changes in the outcome (PA) are attributable the mediator, and the simultaneous test of both represents an evaluation of the extent to which the intervention effect was mediated by the mechanisms hypothesized to cause changes in PA. A liberal coding for each theory was applied where support for a test was obtained for at least one construct/behaviour at one time point. This was deemed a valid assumption because all of the above noted theories/models are multivariate in nature and do not stipulate that all of their constructs necessarily function in tandem. Decision procedures were based on significant/null findings (p < .05) in each study as well as the establishment of at least a small effect size using standardized criteria [5] (*d *> .19; η^2 ^> .009).

Narrative appraisal and evidence synthesis were subsequently performed [24]. Key factors for consideration in this qualitative appraisal included the success or failure of the action, theory, and simultaneous tests, as well as prior review results [9]. Typical interpretations of risk versus harm in outcome research [24] do not translate to the topic of mediators perfectly; evidence was thus classified by 1) weak or no evidence for mediation, 2) mixed evidence for mediation, or 3) strong evidence for mediation.

## Results

**Figure 1:**
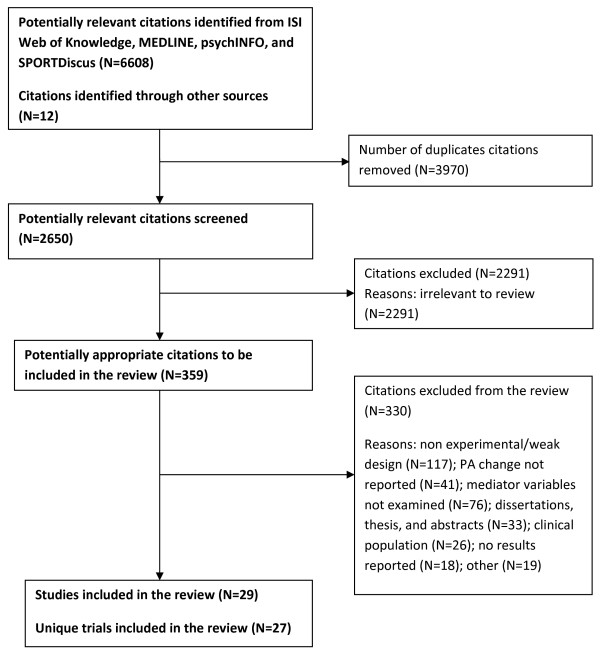
**Results of the Literature Search**.

The literature search yielded a total of 6620 potentially relevant records. Of these, 359 abstracts and full text reports were obtained and reviewed. Twenty nine studies describing 27 unique trials passed the eligibility criteria and were therefore included [25-52] (see Figure 1 based on QUOROM/PRISM guidelines [53]). These 27 trials were not included in the prior reviews on this topic [3,9].

### Study characteristics

The 27 trials examined different types of interventions on physical activity behaviour (see Tables 1 and Additional file 3). In terms of quality rating, five trials were identified as low quality [33,39,42,45,54] and were not subsequently included in the analyses (see Additional file 5). Of the remaining 22 trials, one was scored as high quality [44] and all others were deemed of moderate quality. Most studies used constructs from a chosen theory with intentions of increasing the participants' PA behaviour. The design of the interventions were either randomized control (N = 16), two group experimental (N = 1), quasi experimental (N = 4), stratified control trial (N = 1), non random assignment (N = 1), or pre-post test (N = 4). Trials ranged from two to eight arms, with the majority using a two or three arm design comparing a high theoretical fidelity intervention to a standard public health intervention group (N = 20). Six other two arm studies compared a high fidelity intervention to a control group. The settings of the studies included universities (N = 3), general practice (N = 4), worksites (N = 3), and community settings (N = 3). Sample size ranged from 44 to 31,420. Participants were of both genders (N = 20), or women only (N = 7). Physical activity was most commonly assessed using IPAQ (N = 3), 7 day PAR (N = 8), and GLTEQ (N = 6). The interventions were based on SCT (N = 3), TTM (N = 9), TPB (N = 3), SDT (N = 2), and PMT (N = 2) among others. The interventions ranged in length from two weeks to 24 months. Nine studies had follow up tests from one month to one year after the end of the intervention. The follow up tests ranged from short interventions with long follow up periods (N = 2) to follow up tests with a length approximately equal to the length of the intervention period (N = 7); for example a 6 month intervention with a 6 month follow up. Interventions examined the effects of counselling or group sessions (N = 11), telephone (N = 2) or email reminders (N = 5), print materials (N = 4), a combination (N = 4), and other methods on PA levels and mediating variables. Most studies had a physical activity target set at 30 minutes of moderate intensity activity most days of the week (N = 15). Six studies set a target of three days of activity, either vigorous (N = 2) or moderate (N = 4), while two studies opted for a target of either 20 minutes of vigorous activity for three days per week, or 30 minutes of moderate activity for five days a week.

**Table 1:** Characteristics of Included Trial Reports (N = 27)

Characteristic	Value
Study Design	
Trial, N (%)	
Randomized Control Trial	16 (59)
2 group experimental	1 (3)
Quasi-experimental	4 (14)
Stratified Control Trial	1 (3)
Non random assignment	1 (3)
Pre post test	4 (14)
Arm, N (%)	
3 Arm trial	7 (25)
2 Arm trial	16 (59)
4+ Arm trial	3 (11)
Sample size, median (min, max), N	150 (44, 31,420)
Quality score, median (min, max)	6 (3, 7)
	
Participant population	
	
All Female trial, N (%)	7 (25)
Both gender trial, N (%)	20 (74)
Setting, N (%)	
Practice	4 (14)
Home	2 (7)
Work Site	3 (11)
WIC	1 (3)
University	3 (11)
Community	3 (11)
Not reported	11 (40)
	
Intervention	
	
Theory, N (%)	
SCT	3 (11)
TTM	9 (33)
PMT	2 (7)
SDT	2 (7)
TPB	3 (11)
	
Duration, median (min, max), wk	12 (2, 104)
Follow up test post intervention, N (%)	8 (29)
	
PA target, N (%)	
30 minutes MVPA most days/week	17 (62)
30 minutes MVPA 3 days/week	4 (14)
20 minutes VPA 3 days/week	2 (7)
	
Outcome measures	
	
PA, N (%)	
PAR	7 (25)
GLTEQ	6 (22)
CHAMPS	2 (7)
IPAQ	3 (11)
SQUASH	1 (3)
Objective measure	2 (7)
Other self-report questionnaire	7 (25)
No. of psychological assessment tools, median (min, max)	3 (1, 6)
	
Reporting outcomes	
	
Change in PA behaviour, N (%)	27 (100)
Change in mediators, N (%)	27 (100)
Mediator analysis, N (%)	6 (22)
	
Type of mediation test (N = 6)	
	
Baron and Kenny, N (%)	3 (50)
Unspecified, N (%)	2 (33)
Mackinnon et al., N (%)	2 (33)
Freedman-Schatzkin, N (%)	1 (16)
Bootstrap, N (%)	1 (16)

*note: some studies employed more than one type of test

### General Evidence of Mediation

Of the 22 samples, 11 did not show evidence that the intervention was effective in changing PA [25,26,28,32,36,40,41,43,49-51], thus failing the first consideration in most investigations/analyses of mediators [11]. These studies generally had null results on the proposed mediators as well with only four [25,26,41,51] of the 11 samples demonstrating evidence that the intervention had an action theory link. Of the remaining 11 samples where the intervention demonstrated change in PA [27,29-31,37,38,44,46-48,52], all showed evidence of an action test link, whereby at least some of the proposed mediating constructs changed from the intervention. These were not distinguishable by methodological characteristics. For example, studies ranged from college undergraduates [46,48] to the general population [29,47] or specified populations [30,31]. Proposed mediators included constructs from TTM [47], SCT [30], PMT [46], SDT [35], and TPB[48] and duration of the intervention ranged from two weeks [46] to one year [37]. Further, the interventions for these studies ranged from relatively straightforward messaging [46,48] to more intensive and long term counselling and workshops [30,31], while participants ranged from carefully screened inactive samples [47] to no consideration of baseline physical activity [46] and comparisons were with true controls [48] or generic physical activity intervention groups [47].

Of these 11 studies to show evidence that the intervention could change PA and support the action test link, only five reported a conceptual theory test [27,29,44,47,52], and six reported a mediator test [27,29,37,44,47,52]. All five conceptual theory tests showed at least some support for a link between a proposed mediating construct and PA change, but the resulting tests of mediation was supported in four [27,29,37,47] of the six samples.

### Evidence of Mediation by Theory

#### Transtheoretical Model

Eight of the 22 samples employed constructs of the TTM as mediators of change (defined as including at least two TTM constructs specified by Prochaska and DiClemente [55]) [28,31,32,34,36,44,47,56]. In all cases the studies were well-controlled designs where the TTM concepts were employed in the interventions. One study, however, did not employ these interventions to standard control or exercise prescription [36] and should be noted as deviant from the other eight studies. Furthermore, four of these eight studies reported a null effect of the intervention on PA change [28,32,36,49] and subsequent null action theory tests on TTM constructs. The remaining four studies [31,44,47,52], however, all had evidence of at least one TTM construct showing an action theory link. Three of these studies tested for a conceptual theory link with significant evidence for at least one TTM construct and these three studies also employed mediation effect tests [44,47,52]. Interestingly, two of these studies demonstrated that TTM mediators failed to attenuate the relationship between the intervention and behaviour [44,52]. By contrast, Napolitano et al. [47] demonstrated that behavioural processes of change (and cognitive processes as a suppressor) were able to account for the relationship between the intervention and behaviour in a formal mediation test. Taken together, the TTM currently has mixed results in terms of intervention efficacy and in tests of mediation of its constructs.

#### Social Cognitive Theory

Three studies have tested SCT (defined as including at least two constructs as specified by Bandura [57]) [30,37,51]. Two of the studies followed controlled trials [30,51], while the other employed a quasi-experimental design [37]. Furthermore, one [51] of these three studies did not support the effect of the intervention on behaviour although all studies did have some support for an action theory link. None of the studies tested for a conceptual theory link, but Hallam and Petosa [37] provided evidence that self-regulation was a mediator of behaviour at 12 months post-intervention, but did not show support for self-efficacy or outcome expectations. It should be noted that this mediation relationship was also inconsistent and not present at six weeks or six month assessments and it did not examine mediation using product-of coefficient tests recommended by MacKinnon and colleagues [10]. Thus, there is evidence for possible mediation between selected SCT constructs and intervention-PA change but the available studies are extremely limited and mixed at present.

#### Theory of Planned Behaviour

Three studies have employed the TPB (defined as including at least two constructs as specified by Ajzen [18]) [40,48,50]. The methods for these studies include two experimental persuasive communication interventions among undergraduates [40,48] and one quasi-experimental community design [50]. Two of these studies, however, show null results in terms of a link between the intervention and PA as well as the action theory test for a link between the intervention and TPB constructs [40,50]. The single study [48] to show support for an effect of the intervention on changes in PA demonstrated action theory links with intention, perceived behavioural control, and affective attitude (dependent on baseline values) yet no conceptual theory test formal mediation analysis was performed. Overall, the evidence is too limited from a paucity of research and lack of actual behaviour change in the interventions to make a judgement of the effectiveness of TPB as a mediator in PA interventions.

#### Protection Motivation Theory

Two studies have applied PMT (defined as including at least two constructs as specified by Rogers [19]) [46,56]. Plotnikoff et al. [56], were unable to show effects of their work site intervention on the proposed mediators or behaviour, thus failing to support the action theory test and the intervention-PA link. Milne et al. [46] showed that their intervention had an effect on short-term PA change and supported the action theory link for all PMT constructs in a sample of undergraduate students although no formal conceptual theory test and mediation analyses were conducted. Obviously the limited applications of PMT warrant more research.

#### Self-Determination Theory

Two studies have employed SDT (defined as including at least two constructs as specified by Deci and Ryan [14]) in interventions using randomized experimental designs in community samples [35,43]. Both studies employed interventions tailored to the concepts of SDT. Levy and Cardinal [43] employed a print mail-out intervention and did not show changes in SDT constructs or behaviour, thus failing to support the action theory test and the link of the intervention to PA. By contrast, Fortier et al. [35], used a primary care intervention setting and showed the intervention had an effect on behaviour and an action theory link for SDT constructs of autonomy (motivation and support) but not competence. The investigators also reported support for a conceptual theory link between autonomy support and PA but no formal tests of the mediation effect was implemented. More research is needed to evaluate SDT as a mediator of behaviour given these limited findings.

### Evidence of Mediation by Construct

#### Self-Efficacy/Perceived Control

Nineteen of the 22 studies employed a self-efficacy type construct, defined as an appraisal of confidence or capability to perform physical activity. Of these, nine had null effects of the intervention on PA change [25,26,28,32,36,40,49-51] and only two of these showed support for subsequent action theory tests [25,26]. Among the remaining 10 studies to support the initial intervention-PA link, seven supported a significant action theory test for the effect of the intervention on changes in self-efficacy/control [27,30,38,46-48,52]. Four of these seven also reported conceptual model tests [27,44,47,52] and three supported a link between changes in self-efficacy/control and changes in PA [27,44,52]. Of the five studies that employed a formal mediation analysis [27,37,44,47,52], however, only one showed significant support for self-efficacy [27]. In this case, Blanchard et al. [27] demonstrated that task self-efficacy (efficacy to physically perform the behaviour) was a significant mediator of behaviour change but barrier self-efficacy (confidence to overcome hassles) was not. Thus, self-efficacy has considerably limited support for its role as a mediator of PA changes due to interventions at present.

#### Outcome Expectations

Fourteen of the 22 studies reviewed included outcome expectations, defined broadly as expected/anticipated consequences from behavioural or lack of behavioural engagement, as potential mediating constructs [28,30,32,36,37,40,44,46-52]. Of these, seven showed null effects for the intervention on PA [28,32,36,40,49-51] and all but one of these [51] also reported non-significant effects for the action theory test. Of the remaining seven studies, all but one [44] showed support for the action theory test of the intervention's effectiveness in changing outcome expectations. It is interesting to note that many of these studies measured and targeted outcome expectations underlying the affective domain in the intervention (i.e., enjoyment, pain, fear) as opposed to more instrumental and distal outcome expectations (i.e., weight loss, fitness, chronic disease). Indeed, Parrott et al. [48] showed a significant action theory test with affective outcome expectations but a non-significant action theory test with instrumental outcome expectations when measured separately. Only three studies, however, reported subsequent conceptual theory tests [44,47,52], and the four tests to examine the mediation effect all reported non-significant findings for outcome expectation constructs [37,44,47,52]. Overall, there is limited evidence for outcome expectations as a mediator of PA interventions. Distinctions by affective/proximal and instrumental/distal expectations in action theory tests suggest there may be more evidence for the affective/proximal domain in mediation but these studies did not report conceptual theory tests or specific mediated effects.

#### Self-Regulatory Processes

Defined generally as planning, scheduling, and self-organizational behaviours, self-regulatory processes were measured in some capacity in 16 of the 22 studies [28,31,32,34,36-38,40,41,44,46-48,50,51,58]. Eight of these studies showed null effects for the intervention on behaviour change [28,32,36,40,41,49-51] and only two of these had significant action theory tests [41,51] suggestive of generally null/ineffective trials. Of the remaining eight studies, however, six reported evidence of significant action theory tests [37,44,46-48,52]. For example, Milne et al. [46] showed that planning/implementation intentions affected increases in behaviour beyond those of an intervention that increased self-efficacy and outcome expectations. Despite these supportive action theory tests, only three studies reported conceptual theory tests, although all provided support for self-regulatory constructs [44,47,52]. Finally, of the four tests to examine a mediated effect, Hallam and Petosa [37] and Napolitano et al. [47], showed that changes in self-regulation (via self-regulation and behavioural processes of change respectively) mediated the relationship between the intervention and changes in PA. Still, there were two studies that demonstrated no mediation of a successful intervention through self-regulation processes (behavioural processes) [34,44]. Overall, there is some evidence for mediation between self-regulation processes and behaviour but results are mixed.

#### Social Constructs

Variables with social referents typically encompassed either subjective norm (perceived pressure to perform the behaviour) or social support (support from others to perform the behaviour). Nine studies employed such variables in these studies [29,34,35,39,40,43,48,50,51] but five of these studies did not show support for the effectiveness of the intervention on changes in PA [40,43,49-51] nor did they demonstrate significant action theory tests on the social constructs. Three of the remaining four studies showed significant action and conceptual theory tests and it is notable that all three studies contain support rather than normative constructs [29,35,52]. Tests of the mediated effect, however, were conducted among two of these studies and the results were mixed. Specifically, Cerin and colleagues [29] demonstrated mediation while Fahrenwald et al. [34] did not show evidence for the mediation capacity of social support. Thus, social constructs have some evidence for mediation of PA interventions and behaviour but results are limited and positive findings have only been with support, not norms.

## Discussion

Theories of PA behaviour suggest that particular constructs are critical antecedents of behavioural engagement. These constructs are hypothesized as components of a causal chain, suggesting that if the mediators are changed, behaviour change should follow [6]. Early reviews based on theoretical mediators of behaviour change, however, suggested that few formal tests of mediation had been conducted and limited evidence was available to support this proposition [3,9]. Therefore, the purpose of this review was to provide an update of the literature on PA interventions that have included proposed mediators of behaviour, focusing specifically on primary prevention in adults since the time of these prior reviews.

The review yielded 29 studies from 27 independent samples to appraise our current understanding of PA mediators in interventions. Five studies were omitted from the analyses due to low quality but the other 22 trials showed moderate (n = 21) or high (n = 1) quality and thus relatively low risk of bias. Almost all studies did not meet the category for high quality because they failed to include a direct measure of physical activity behaviour and did not report on a pilot intervention to demonstrate that it could affect the mediators. Otherwise, the 22 trials generally showed many high quality features such as random assignment, a theoretical-base, reliable and valid measures of the mediators, and reliable measures of self-reported PA.

Overall, 11 studies showed that the intervention had an effect on PA behaviour change and all of these studies subsequently had an action theory link [59]. That is, all 11 studies showed some evidence that the intervention also changed the proposed mediators. By contrast, a conceptual theory link [59] was seldom reported (5/11 studies). Conceptual theory links demonstrate that changes in the mediators are related to the PA outcome. These are often the foundation for using a theory or mediator construct before initiation of the intervention [11], but future work needs to test this link regularly in reported trials with mediators. Formal tests of mediation were also only conducted in six of the 11 cases where the procedure may have been appropriate (i.e., intervention effect on behaviour, evidence of action theory link, conceptual theory link or probable conceptual theory link). In terms of behavioural mediation by theory, TTM, SCT, TPB, PMT, and SDT all showed some evidence for action theory tests and all have shown evidence for conceptual theory tests in the past, but only the TTM employed tests of a mediated effect of its constructs. The results, when divided by theory, are too limited in number to make particular judgements at present.

A division at the construct level [6,12,18,20], however, provides a larger sample for assessment. Self-regulation constructs (e.g., planning, behavioural processes) from trials where the intervention changed PA behaviour showed 75% (6 of 8 studies) support for action theory tests and all three of the conceptual theory tests conducted were significant. Mediated effect tests of the construct, however, were mixed with two showing support and two not providing evidence for mediation. Our appraisal of self-regulation is similar to the original comments made by Lewis et al. [9]; the construct has the most support thus far but still demonstrates mixed findings. Still, it seems prudent to include a self-management and self-regulatory component to PA interventions.

Results of self-efficacy and outcome expectation-type constructs as mediators were weak or limited. Self-efficacy constructs among intervention studies that affected PA change showed relatively strong evidence for action theory (7 of 10 studies) and conceptual theory (3 of 4 studies reported) links, but a mediated effect was not supported in four of the five formal tests conducted. Outcome expectation constructs had similar results in terms of evidence for an action theory link (6/7 studies) but zero of the four tests for a mediated effect were significant. There was some notable differences between affective and instrumental outcome expectations (see [60] for extended commentary) with positive changes in affective outcome expectations linked to positive changes in behaviour more than instrumental outcome expectations. Still, the relatively few studies on this topic and absence of any formal mediation tests render this point as speculative at present.

Social constructs were limited to only four studies where the intervention had produced significant changes in PA; however, three of these four studies showed an action theory link. Social support was also a mediator of behaviour change in one formal test of mediation, but was unable to show a mediated effect in the only other test with this construct. There was no evidence for the mediation capacity of subjective norm. Although limited literature precludes any definitive conclusions, social constructs, particularly social support, may have utility as mediators of change but findings are mixed at present.

A key finding of the review, however, was that half the interventions failed to change both behaviour and the proposed mediators through the action theory link. This does not challenge the internal structure of our leading theories and constructs at present as much as demonstrate that our interventions are generally ineffective. To evaluate the mediation capacity of a theory, the behavioural link and action link are important first steps in mediation [11]. Pilot studies showing evidence that the intervention can change the proposed mediators are recommended in future research before large-scale trials are conducted.

The poor performance of PA interventions has been duly recognized [3,4], and it is much easier to comment on this problem than to provide solutions. Nevertheless, it is important to provide some commentary on this issue. A most pragmatic possibility for these results may be attenuation from measurement error. For example, indirect (self-report) PA measures featured in these studies may lack the sensitivity to distinguish change between the groups and the psychological constructs may equally lack precision [3]. Direct measures of PA are recommended in future trials. Still, this seems unlikely to be the sole reason for these null effects; many of the studies were able to demonstrate time effects (i.e., main effects), and the proposed mediators generally show moderate to large bivariate correlations with PA in prediction tests [e.g., [61]].

Clearly more innovation and higher fidelity interventions are needed. In the studies reviewed, there was a very similar genre of intervention. These typically focused on a persuasive educational component about the benefits of PA and hazards of inactivity followed by problem solving suggestions to regulate action and overcome barriers. Although this approach could be helpful to some, it was not helpful to change proposed mediators in over 50% of the cases reviewed and these null results were not readily identifiable as discrepant intervention styles from successful trials. The problem occurring may be that the approach is an insufficient band-aid to overcome the real-world obstacles and different values that some inactive participants experience. Social and environmental structures may be so grounded and geared to sedentary lifestyles that individual-level, inexpensive, patches may not resonate with the inactive populace [41]. The limits of these "downstream" approaches have been recognized [62]. Approaches at system-level social and environmental change may be needed to aid many people [63]. This approach, of course, is costly and does not lend itself to the tight-budget three-year RCT; indeed, it is likely to conflict with other societal and industrial aims.

Interacting with these more systemic social and environmental issues may be systemic internal issues. Enacting a potentially fatiguing, boring, and time-consuming behaviour on a repeated basis in the face of other behavioural options and values is likely to pose an enormous daily challenge to many people. Some of this may arise from differences in genetic predisposition and other individual differences that are not easily intervened upon [64], while some of these decisions may be the result of informed free will.

When considering these possibilities, it seems important for future interventions to become more innovative and target proposed mediators with a higher fidelity. Using the tenets of SCT as a guide[57], the experiential qualities of the behaviour seem the most telling way to affect cognitions rather than passive approaches. Experiences of valued personal outcomes (e.g., enjoyment, pleasure, satisfaction) and behavioural control/self-efficacy, through shifts in behavioural, environmental and social experiences of PA may be the most effective intervention alongside increasing self-regulatory skills. At this time, we recommend that interventions focus on altering the behavioural experience in an attempt to improve fidelity and affect change in proposed mediators.

It is important to highlight the limitations of this review in order to provide a context for the results. First, the assessment is limited to published work and may be subject to publication bias. Given the high rate of null effects in these results, the bias may be minimal but no formal test of publication bias can be conducted. Second, the work contained in this review is limited to English written journals and thus the results cannot generalize to studies conducted and published in other languages. Finally, the review is limited to the search terms and data-bases contained in our methods section, which followed the precedent of Baranowski et al. [3] and Lewis et al. [9]. Studies that have not been abstracted with these key words will be missing from our review.

## Conclusions

In summary, less than half of the 22 studies reviewed showed evidence that the intervention changed PA and the proposed meditating constructs of behaviour. Among the studies to show these effects, about half subsequently performed tests of the mediating effect or that changes in the proposed mediator were linked to changes in PA. Tests of mediated effect also showed mixed outcomes. Differences by theory were not discernable at this time, but self-regulation constructs had the most evidence for mediation. The general null findings of many behavioural interventions are a timely concern. Innovation and increased fidelity of interventions is needed and should be a priority for future research.

## Abbreviations

SD: Standard Deviation; IPAQ: International Physical Activity Questionnaire; 7 day PAR: 7 day Physical Activity Recall; GLTEQ: Godin Leisure Time Exercise Questionnaire; RCT: Randomized Control Trial; ES: Effect Size.

## Competing interests

The authors declare that they have no competing interests.

## Authors' contributions

RR conceived the review, participated in the coordination of the search, assessed study quality, analyzed and interpreted the results, and drafted the majority of the manuscript. LP carried out the literature search, participated in the study quality assessment, compiled the results into tables, and drafted the methods section. Both authors read and approved the final manuscript.

## Authors' information

RR, PhD, holds a Canadian Institutes for Health Research New Investigator Award and is currently an Associate Professor in the School of Exercise Science, Physical and Health Education at the University of Victoria.

LP, BPE/BEd, MA, is a Research Associate in the Behavioural Medicine Laboratory at the University of Victoria.

## Supplementary Material

Additional file 1 **Excluded articles**. This file contains the list of articles that did not meet our inclusion criteria. The articles are grouped based on the criteria the articles failed to meet. Click here for file

Additional file 2 **Search syntax**. This file contains the search terms used in this review. Click here for file

Additional file 3 **Review Table of proposed mediators and physical activity behavior**. This file contains data extracted from each included article in table form for quick reference. Click here for file

Additional file 4 **Data extraction**. This file contains the data extracted from each included article. Click here for file

Additional file 5 **Quality of studies using tool developed by Lubans, Foster, and Biddle (2008)**. This file contains the tool used to assess the quality of each study included in the review. Each study was assessed by a series of questions listed below. BMI: Body Mass Index; RCT: Randomized control trial; PA: physical activity; MI: motivational interviewing; ES: effect size; OR: odds ratio; PAR: physical activity recall. TTM: transtheoretical model; POC: processes of change; SCT: social cognitive theory; IPAQ: International Physical Activity Questionnaire; GLTEQ: Godin Leisure Time Exercise Questionnaire; SDT: self-determination theory; PMT: protection motivation theory; TPB: theory of planned behaviour; PCB: perceived behavioural control; CHD: coronary heart disease. Click here for file

## References

[B1] Warburton DER Katzmarzyk PT Rhodes RE Shephard RJ Evidence-informed physical activity guidelines for Canadian adults Applied Physiology, Nutrition and Metabolism 2007 32 S16 S68 10.1139/H07-123 18213940

[B2] Canadian Fitness and Lifestyle Research Institute 2002 Physical Activity Monitor http://www.cflri.ca/eng/statistics/surveys/pam2002.php

[B3] Baranowski TC Anderson Carmack C Mediating variable framework in physical activity interventions: How are we doing? How might we do better? American Journal of Preventive Medicine 1998 15 266 297 10.1016/S0749-3797(98)00080-4 9838973

[B4] Hillsdon M Foster C Thorogood M Interventions for promoting physical activity Cochrane Database of Systematic Reviews 2005 1 10.1002/14651858.CD003180.pub2 PMC4164373 15674903

[B5] Cohen J A power primer Psychological Bulletin 1992 112 155 159 10.1037/0033-2909.112.1.155 19565683

[B6] Noar SM Zimmerman RS Health behavior theory and cumulative knowledge regarding health behaviors: are we moving in the right direction? Health Education Research 2005 20 275 290 10.1093/her/cyg113 15632099

[B7] Sheeran P Hewstone M, Stroebe W Intention-behaviour relations: A conceptual and empirical review European Review of Social Psychology 2002 Chichester, UK: John Wiley & Sons 1 36 10.1080/14792772143000003

[B8] Weinstein ND Misleading tests of health behavior theories Annals of Behavioral Medicine 2007 33 1 10 10.1207/s15324796abm3301_1 17291165

[B9] Lewis BA Marcus BH Pate RR Dunn AL Psychosocial mediators of physical activity behavior among adults and children American Journal of Preventive Medicine 2002 23 Suppl 2 6 35 12133735 10.1016/s0749-3797(02)00471-3

[B10] Mackinnon DP Lockwood CM Hoffman JM West SG Sheets V A comparison of methods to test mediation and other intervening variable effects Psychological Methods 2002 7 83 103 10.1037/1082-989X.7.1.83 11928892 PMC2819363

[B11] Cerin E Mackinnon DP A commentary on current practice in mediating variable analyses in behavioural nutrition and physical activity Public Health Nutrition 2008 12 1182 1188 10.1017/S1368980008003649 18778534 PMC4207270

[B12] Bandura A Health promotion from the perspective of social cognitive theory Psychology and Health 1998 13 623 649 10.1080/08870449808407422

[B13] Prochaska JO Velicer WF The transtheoretical model of health behavior change American Journal of Health Promotion 1997 12 38 48 10170434 10.4278/0890-1171-12.1.38

[B14] Deci EL Ryan RM Intrinsic motivation and self-determination in human behavior 1985 New York: Plenum Press

[B15] Ajzen I Driver BL Prediction of leisure participation from behavioral, normative, and control beliefs: An application of the theory of planned behavior Leisure Sciences 1991 13 185 204 10.1080/01490409109513137

[B16] Baron RM Kenny DA The moderator-mediator variable distinction in social psychological research: Conceptual, strategic, and statistical considerations Journal of Personality & Social Psychology 1986 51 1173 1182 10.1037/0022-3514.51.6.1173 3806354

[B17] Bandura A Health promotion by social cognitive means Health Education and Behavior 2004 31 143 164 10.1177/1090198104263660 15090118

[B18] Ajzen I The theory of planned behavior Organizational Behavior and Human Decision Processes 1991 50 179 211 10.1016/0749-5978(91)90020-T

[B19] Rogers RW Cacioppo JT, Petty RE Cognitive and physiological processes in fear appeals and attitude change: A revised theory of protection motivation Social Psychophysiology 1983 New York: Guilford Press 153 176

[B20] Fishbein M Triandis HC Kanfer FH Becker M Middlestadt SE Eichler A Baum A, Revenson TA Factors influencing behavior and behavior change Handbook of health psychology 2001 Mahwah, New Jersey: Lawrence Erlbaum Associates 3 17

[B21] Lubans DR Foster C Biddle SJH A review of mediators of behavior in interventions to promote physical activity among children and adolescents Preventive Medicine 2008 47 463 470 10.1016/j.ypmed.2008.07.011 18708086

[B22] Cerin E Barnett A Baranowski T Testing theories of dietary behavior change in youth using the mediating variable model with intervention programs Journal of Nutrition Education and Behavior 2009 41 309 318 10.1016/j.jneb.2009.03.129 19717113

[B23] Higgins JPT Green S Cochrane Handbook for Systematic Reviews of Interventions Vol. Version 5.0.1 2008 The Cochrane Collaboration

[B24] Grade Working Group Grading quality of evidence and strength of recommendations British Medical Journal 2004 328 1490 1498 10.1136/bmj.328.7454.1490 15205295 PMC428525

[B25] Ash S Reeves M Bauer J Dover T Vivanti A Leong C O'Moore Sullivan T Capra S A randomised control trial comparing lifestyle groups, individual counselling and written information in the management of weight and health outcomes over 12 months International Journal of Obesity 2006 30 1557 1564 10.1038/sj.ijo.0803263 16534529

[B26] Bennett JA Young HM Nail LM Winters-Stone K Hanson G A telephone-only motivational intervention to increase physical activity in rural adults Nursing Research 2008 57 24 32 10.1097/01.NNR.0000280661.34502.c1 18091289

[B27] Blanchard CM Fortier M Sweet S O'Sullivan T Hogg W Reid RD Sigal RJ Explaining physical activity levels from a self-efficacy perspective: The physical activity counselling trial Annals of Behavioral Medicine 2007 34 323 328 10.1007/BF02874557 18020942

[B28] Bock B Marcus BH Pinto BM Forsyth LH Maintenance of physical activity following an individualized motivationally tailored intervention Annals of Behavioral Medicine 2001 23 79 87 10.1207/S15324796ABM2302_2 11394558

[B29] Cerin E Taylor L Leslie E Owen N Small-scale randomized controlled trials need more powerful methods of mediational analysis than the Baron-Kenny method Journal of Clinical Epidemiology 2006 59 457 464 10.1016/j.jclinepi.2005.11.008 16632133

[B30] Cramp AG Brawley LR Moms in motion: A group-mediated cognitive-behavioral physical activity intervention International Journal of Behavioral Nutrition and Physical Activity 2006 3 1479 10.1186/1479-5868-3-23 PMC1564034 16925809

[B31] Dallow CB Anderson J Using self-efficacy and a transtheoretical model to develop a physical activity intervention for obese women American Journal of Health Promotion 2003 17 373 381 12858617 10.4278/0890-1171-17.6.373

[B32] Dinger MK Heesch K Cipriani G Qualls M Comparison of two email-delivered, pedometer-based interventions to promote walking among insufficiently active women Journal of Science and Medicine in Sport 2007 10 297 302 10.1016/j.jsams.2006.07.011 16950654

[B33] Elbel R Aldana S Bloswick D Lyon JL A pilot study evaluating a peer led and professional led physical activity intervention with blue-collar employees Work 2003 21 199 210 14600324

[B34] Fahrenwald NL Atwood JR Noble Walker S Johnson DR Berg K A randomized pilot test of "moms on the move": A physical activity intervention for WIC mothers Annals of Behavioral Medicine 2004 27 82 90 10.1207/s15324796abm2702_2 15026292

[B35] Fortier MS Sweet SN O'Sullivan TL Williams GC A self-determination process model of physical activity adoption in the context of a randomized controlled trial Psychology of Sport and Exercise 2007 8 741 757 10.1016/j.psychsport.2006.10.006

[B36] Gallagher KI Jakicic JM Napolitano MA Marcus BH Psychosocial factors related to physical activity and weight loss in overweight women Medicine and Science in Sports and Exercise 2006 38 971 980 10.1249/01.mss.0000218137.25970.c6 16672853

[B37] Hallam JS Petosa R The long-term impact of a four-session work-site intervention on selected social cognitive theory variables linked to adult exercise adherence Health Education and Behavior 2004 31 88 100 10.1177/1090198103259164 14768660

[B38] Hurling R Catt M De Boni M Fairley BW Hurst T Murray P Richardson A Singh Sodhi J Using internet and mobile phone technology to deliver an automated physical activity program: Randomized controlled trial Journal of Medical Internet Research 2007 9 e7 10.2196/jmir.9.2.e7 17478409 PMC1874722

[B39] Jacobs AD Ammerman AS Ennett ST Campbell MK Tawney KW Aytur SA Marshall SW Will JC Rosamond WD Effects of a tailored follow-up intervention on health behaviors, beliefs, and attitudes Journal of Women's Health 2004 13 557 568 10.1089/1540999041281016 15257847

[B40] Jones LW Sinclair RC Rhodes RE Courneya KS Promoting exercise behaviour: An integration of persuasion theories and the theory of planned behaviour British Journal of Health Psychology 2004 9 505 521 10.1348/1359107042304605 15509358

[B41] Kinmonth AL Wareham NJ Hardeman W Sutton S Prevost AT Fanshawe T William KM Ekelund U Spiegelhalter D Griffin SJ Efficacy of a theory-based behavioural intervention to increase physical activity in an at-risk group in primary care (ProActive UK): A randomised trial Lancet 2008 371 41 48 10.1016/S0140-6736(08)60070-7 18177774

[B42] Kloek GC van Lenthe FJ van Nierop PWM Koelen MA Mackenbach JP Impact evaluation of a Dutch community intervention to improve health related behaviour in deprived neighbourhoods Health and Place 2006 12 665 677 10.1016/j.healthplace.2005.09.002 16253541

[B43] Levy SS Cardinal BJ Effects of a self-determination theory-based mail-mediated intervention on adults' exercise behavior American Journal of Health Promotion 2004 18 345 349 15163133 10.4278/0890-1171-18.5.345

[B44] Lewis BA Forsyth LH Pinto BM Bock BC Roberts M Marcus BH Psychosocial mediators of physical activity in a randomized controlled intervention trial Journal of Sport and Exercise Psychology 2006 28 193 204

[B45] Little P Doward M Gralton S Hammerton L Pillinger J White P Moore M McKenna J Payne S A randomized controlled trial of three pragmatic approaches to initiate increased physical activity in sedentary patients with risk factors for cardiovascular disease British Journal of General Practice 2004 54 189 195 15006124 PMC1314829

[B46] Milne S Orbell S Sheeran P Combining motivational and volitional interventions to promote exercise participation: Protection motivation theory and implementation intentions British Journal of Health Psychology 2002 7 163 184 10.1348/135910702169420 14596707

[B47] Napolitano MA Papandonatos GD Lewis BA Whiteley JA Williams DM King AC Bock BC Pinto B Marcus BH Mediators of physical activity behavior change: A multivariate approach Health Psychology 2008 27 409 418 10.1037/0278-6133.27.4.409 18642998 PMC2692944

[B48] Parrott MW Tennant LK Olejnik S Poudevigne MS Theory of planned behavior: Implications for an email-based physical activity intervention Psychology of Sport and Exercise 2008 9 511 526 10.1016/j.psychsport.2007.07.002

[B49] Plotnikoff RC Brunet S Courneya K Spence J Birkett N Marcus B Whitely J The efficacy of stage-matched and standard public health materials for promoting physical activity in the workplace: The Physical Activity Workplace Study (PAWS) American Journal of Health Promotion 2007 21 501 509 17674637 10.4278/0890-1171-21.6.501

[B50] Reger B Cooper L Booth-Butterfield S Smith H Bauman A Wootan M Middlestadt S Marcus B Greer F Wheeling walks: a community campaign using paid media to encourage walking among sedentary older adults Preventive Medicine 2002 35 285 292 10.1006/pmed.2002.1074 12202072

[B51] Rovniak LS Hovell MF Wojcik JR Winett RA Martinez-Donate AP Enhancing theoretical fidelity: An e-mail-based walking program demonstration American Journal of Health Promotion 2005 20 85 95 16295700 10.4278/0890-1171-20.2.85

[B52] Fahrenwald NL Atwood JR Johnson DR Mediator analysis of moms on the move Western Journal of Nursing Research 2005 27 271 291 10.1177/0193945904273275 15781903

[B53] Moher D Cook DJ Eastwood S Olkin I Rennie D Stroup DF Improving the quality of reports of meta-analyses of randomised controlled trials: The QUOROM statement Lancet 1999 354 1896 1900 10.1016/S0140-6736(99)04149-5 10584742

[B54] Cardinal BJ Spaziani MD Effects of Classroom and Virtual "Lifetime Fitness for Health" Instruction on College Students' Exercise Behavior Physical Educator 2007 64 205 213

[B55] Prochaska JO DiClemente CC Transtheoretical therapy: Toward a more integrative model of change Psychotherapy: Theory, Research & Practice 1982 19 276 288 10.1037/h0088437

[B56] Plotnikoff RC McCargar L Wilson PM Loucaides CA Efficacy of an email intervention for the promotion of physical activity and nutrition behavior in the workplace context American Journal of Health Promotion 2005 19 422 429 16022206 10.4278/0890-1171-19.6.422

[B57] Bandura A Self-efficacy, the exercise of control 1997 New York: Freeman

[B58] Plotnikoff RC Blanchard C Hotz SB Rhodes R Validation of the decisional balance scales in the exercise domain from the transtheoretical model: A longitudinal test Measurement in Physical Education and Exercise Science 2001 5 191 206 10.1207/S15327841MPEE0504_01

[B59] Chen HT Theory-Driven Evaluations 1990 Newbury Park, CA: Sage

[B60] Rhodes RE Fiala B Conner M Affective judgments and physical activity: A review and meta-analysis Annals of Behavioral Medicine 2009 38 3 180 204 10.1007/s12160-009-9147-y 20082164

[B61] Symons Downs D Hausenblas HA Exercise behavior and the theories of reasoned action and planned behavior: A meta-analytic update Journal of Physical Activity and Health 2005 2 76 97

[B62] McKinlay JB Marceau LD To boldly go... American Journal of Public Health 2000 90 25 33 10.2105/AJPH.90.1.25 10630133 PMC1446117

[B63] Sallis JF Owen N Glanz K, Lewis FM, Rimer BK Ecological models Health Behavior and Health Education 1997 San Francisco:Jossey-Bass 403 424

[B64] Rhodes RE The built-in environment: The role of personality with physical activity Exercise and Sport Sciences Reviews 2006 34 83 88 10.1249/00003677-200604000-00008 16672806

