# Supplementary material for: Mediators of physical activity behaviour change among adult non-clinical populations: a review update
Source: Int J Behav Nutr Phys Act. 2010 May 11;7:37. doi: 10.1186/1479-5868-7-37 (PMC2876989; doi:10.1186/1479-5868-7-37)
Supplement: Additional file 1 — Excluded articles. This file contains the list of articles that did not meet our inclusion criteria. The articles are grouped based on the criteria the articles failed to meet. [file 1479-5868-7-37-S1.DOC]

**Additional file 1**

Title: Excluded articles

Description: This file contains the list of articles that did not meet our inclusion criteria. The articles are grouped based on the criteria the articles failed to meet.

Non experimental design

Allan, J. D. (2003). Commentary by Allan: 'evaluation of individually tailored interventions on exercise adherence'. *Western Journal of Nursing Research, 25*(6), 644-646.

Annesi, J. J. (2004). Relationship of social cognitive theory factors to exercise maintenance in adults. *Perceptual and Motor Skills, 99*(1), 142-148.

Armitage, C. J. (2005). Can the theory of planned behavior predict the maintenance of physical activity? *Health Psychology, 24*(3), 235-245.

Baranowski, T., Anderson, C., & Carmack, C. (1998). Mediating variable framework in physical activity interventions. how are we doing? how might we do better? *American Journal of Preventive Medicine, 15*(4), 266-297.

Bauman, A. E., Bellew, B., Owen, N., & Vita, P. (2001). Impact of an Australian mass media campaign targeting physical activity in 1998. *American Journal of Preventive Medicine, 21*(1), 41-47.

Bauman, A. E., Sallis, J. F., Dzewaltowski, D. A., & Owen, N. (2002). Toward a better understanding of the influences on physical activity: The role of determinants, correlates, causal variables, mediators, moderators, and confounders. *American Journal of Preventive Medicine, 23*(2), 5-14.

Besculides, M., Zaveri, H., Hanson, C., Earns, R., Gregory-Mercado, K., & Will, J. (2008). Best practices in implementing lifestyle interventions in the WISEWOMAN program: Adaptable strategies for public health programs. *American Journal of Health Promotion, 22*(5), 322-328.

Betterley, C. (2004). Eatfit intervention. *Journal of Nutrition Education & Behavior, 36*(6), 342-343.

Bickmore, T., Gruber, A., & Picard, R. (2005). Establishing the computer-patient working alliance in automated health behavior change interventions. *Patient Education and Counseling, 59*(1), 21-30.

Booth, M. L., Owen, N., Bauman, A., Clavisi, O., & Leslie, E. (2000). Social-cognitive and perceived environment influences associated with physical activity in older Australians. *Preventive Medicine, 31*(1), 15-22.

Boudreaux, E. D., Wood, K. B., Mehan, D., Scarinci, I., Taylor, C. L. C., & Brantley, P. J. (2003). Congruence of readiness to change, self-efficacy, and decisional balance for physical activity and dietary fat reduction. *American Journal of Health Promotion: AJHP, 17*(5), 329-336.

Bray, S. R. (2007). Self-efficacy for coping with barriers helps students stay physically active during transition to their first year at a university. *Research Quarterly for Exercise and Sport, 78*(2), 61-70.

Buckworth, J., & Wallace, L. S. (2002). Application of the transtheoretical model to physically active adults. *Journal of Sports Medicine & Physical Fitness, 42*(3), 360-367.

Burton, N. W., Turrell, G., Oldenburg, B., & Sallis, J. F. (2005). The relative contributions of psychological, social, and environmental variables to explain participation in walking, moderate-, and vigorous-intensity leisure-time physical activity. *Journal of Physical Activity & Health, 2*(2), 181.

Carmack Taylor, C. L., Boudreaux, E. D., Jeffries, S. K., Scarinci, I. C., & Brantley, P. J. (2003). Applying exercise stage of change to a low-income underserved population. *American Journal of Health Behavior, 27*(2), 99-107.

Cerin, E., Vandelanotte, C., Leslie, E., & Merom, D. (2008). Recreational facilities and leisure-time physical activity: An analysis of moderators and self-efficacy as a mediator. *Health Psychology, 27*(2), S126-s135.

Christensen, U., Schmidt, L., Budtz-Jørgensen, E., & Avlund, K. (2006). Group cohesion and social support in exercise classes: Results from a Danish intervention study. *Health Education & Behavior: The Official Publication of the Society for Public Health Education, 33*(5), 677-689.

Collins, M. A., Barros, M. V. G., Nahas, M. V., Goldfine, B., Lanier, A. B., & Beck, J. (2003). Influence of class size on physical activity behavior changes in a 15-week health-related fitness course. *Revista Portuguesa De Ciencias do Desporto, 3*(1), 24-32.

Collins, R., Lee, R. E., Albright, C. L., & King, A. C. (2004). Ready to be physically active? the effects of a course preparing low-income multiethnic women to be more physically active. *Health Education & Behavior, 31*(1), 47-64.

Conn, V. S., Tripp-Reimer, T., & Maas, M. L. (2003). Older women and exercise: Theory of planned behavior beliefs. *Public Health Nursing, 20*(2), 153-163.

Croteau, K. A., Richeson, N. E., Vines, S. W., & Jones, D. B. (2004). Effects of a pedometer-based physical activity program on older adults' mobility-related self-efficacy and physical performance. *Activities, Adaptation & Aging, 28*(2), 19-33.

Cunningham, G.B. (2007). Development of the physical activity class satisfaction questionnaire. *Measurement in Physical Education and Exercise Science, 11*(3), 161-176,

Dacey, M., Baltzell, A., & Zaichkowsky, L. (2003). Factors in women's maintenance of vigorous or moderate physical activity. *Women in Sport & Physical Activity Journal, 12*(1), 87-111.

Dawson, K. A., & Brawley, L. R. (2000). Examining the relationship between exercise goals, self-efficacy, and overt behavior with beginning exercisers. *Journal of Applied Social Psychology, 30*(2), 315-329.

Dean, M. L. (2001). *Effects of vow-making on adherence to a 12-week personal fitness program, self-efficacy, and theory of planned behavior constructs*. Eugene, OR; United States: Kinesiology Publications, University of Oregon.

Dean, R. N., Farrell, J. M., Kelley, M. L., Taylor, M. J., & Rhodes, R. E. (2007). Testing the efficacy of the theory of planned behavior to explain strength training in older adults. *Journal of Aging and Physical Activity, 15*(1), 1-12.

Deforche, B., & De Bourdeaudhuij, I. (2000). Differences in psychosocial determinants of physical activity in older adults participating in organised versus non-organised activities. *The Journal of Sports Medicine and Physical Fitness, 40*(4), 362-372.

Dinger, M. K., Heesch, K. C., & McClary, K. R. (2005). Feasibility of a minimal contact intervention to promote walking among insufficiently active women. *American Journal of Health Promotion, 20*(1), 2-6.

Droomers, M., Schrijvers, C.T.M., Van de Mheen, H., & Mackenbach, J.P. (1998). Educational differences in leisure-time physical inactivity: A descriptive and explanatory study. *Social Science & Medicine, 24*(11), 1665-1676.

Doshi, A., Patrick, K., Sallis, J. F., & Calfas, K. (2003). Evaluation of physical activity web sites for use of behavior change theories. *Annals of Behavioral Medicine: A Publication of the Society of Behavioral Medicine, 25*(2), 105-111.

Dunn, M. Z. (2008). Psychosocial mediators of a walking intervention among African American women. *Journal of Transcultural Nursing: Official Journal of the Transcultural Nursing Society / Transcultural Nursing Society, 19*(1), 40-46.

Emmons, K.M., Barbeau, E.M., Gutheil, C., Stryker, J.E., & Stoddard, A.M. (2007). Social influences, social context, and health behaviors among working class, multi-ethnic adults. *Health Education & Behavior, 34*(2), 315-344.

Epstein, L. H. (1998). Integrating theoretical approaches to promote physical activity. *American Journal of Preventive Medicine, 15*(4), 257-265.

Faherwald, N.L. & Shangreaux, P. (2006). Physical activity behavior of American Indian Mothers. *Orthopaedic Nursing, 25*(1), 22-29.

Fahrenwald, N. L., & Walker, S. N. (2003). Application of the transtheoretical model of behavior change to the physical activity behavior of WIC mothers. *Public Health Nursing, 20*(4), 307-317.

Fallon, E.A., Wilcox, S., Ainsworth, B.E. (2005). Correlates of self-efficacy for physical activity in African Americans. Women & health, 41(3), 47-62.

Ferney, S. L., & Marshall, A. L. (2006). Website physical activity interventions: Preferences of potential users. *Health Education Research, 21*(4), 560-566.

Fincher, S. B. (2004). *Tailored interventions to increase physical activity and cardiorespiratory fitness in younger black females*. US: ProQuest Information & Learning.

Fischer Aggarwal, B. A., Liao, M., & Mosca, L. (2008). Physical activity as a potential mechanism through which social support may reduce cardiovascular disease risk. *The Journal of Cardiovascular Nursing, 23*(2), 90-96.

Foreyt, J. P., & Poston, W. S.,. (1998). The role of the behavioral counselor in obesity treatment. *Journal of the American Dietetic Association, 98*(10), S27-30.

Gilson, N., McKenna, J., & Cooke, C. (2008). Experiences of route and task-based walking in a university community: Qualitative perspectives in a randomized control trial. *Journal of Physical Activity & Health, 5*, S176-s182.

Guinn, B., Vincent, V., Jorgensen, L., Dugas, D., & Semper, T. (2007). Predicting physical activity among low-income Mexican American women: Application of the theory of planned behavior. *American Journal of Health Behavior, 31*(2), 115-122.

Hagger, M. S., Chatzisarantis, N. L. D., & Biddle, S. J. H. (2002). The influence of autonomous and controlling motives on physical activity intentions within the theory of planned behaviour. *British Journal of Health Psychology, 7*(3), 283.

Haines, D. J., Davis, L., Rancour, P., Robinson, M., Ned-Wilson, T., & Wagner, S. (2007). A pilot intervention to promote walking and wellness and to improve the health of college faculty and staff. *Journal of American College Health, 55*(4), 219-225.

Hammond, S. L., Leonard, B., & Fridinger, F. (2000). The centers for disease control and prevention director's physical activity challenge: An evaluation of a worksite health promotion intervention. *American Journal of Health Promotion, 15*(1), 17-20.

Hardeman, W., Sutton, S., Griffin, S., Johnston, M., White, A., Wareham, N. J., et al. (2005). A causal modelling approach to the development of theory-based behaviour change programmes for trial evaluation. *Health Education Research, 20*(6), 676-687.

Hudec, J. C. (2000). *Individual counseling to promote physical activity*. US: ProQuest Information & Learning.

Hulme, P. A., Walker, S. N., Effle, K. J., Jorgensen, L., McGowan, M. G., Nelson, J. D., et al. (2003). Health-promoting lifestyle behaviors of Spanish-speaking Hispanic adults. *Journal of Transcultural Nursing, 14*(3), 244-254.

Irwin, J. D. (2007). The prevalence of physical activity maintenance in a sample of university students: A longitudinal study. *Journal of American College Health, 56*(1), 37-41.

Ishii, A., Nakiri, M., Nagatomi, K., Tsuji, Y., Hoshiko, M., Yamaguchi, Y., et al. (2007). Effect of a physical activity improvement program using the transtheoretical model at a small-scale company. *The Kurume Medical Journal, 54*(1-2), 1-8.

Jeffries, S. K. (2001). *The role of major and minor stressors in moderating the transtheoretical model of exercise behavior among predominantly low-income patients attending primary care clinics*. US: ProQuest Information & Learning.

Jones, F., Harris, P., Waller, H., & Coggins, A. (2005). Adherence to an exercise prescription scheme: The role of expectations, self-efficacy, stage of change and psychological well-being. *British Journal of Health Psychology, 10*(3), 359-378.

Juniper, K. C., Oman, R. F., Hamm, R. M., & Kerby, D. S. (2004). The relationships among constructs in the health belief model and the transtheoretical model among African-American college women for physical activity. *American Journal of Health Promotion, 18*(5), 354-357.

Karelis, A.D., Fontaine, J. et al. (2008). Psychosocial correlates of cardiorespiratory fitness and muscle strength in overweight and obese post-menopausal women: A MONET study. *Journal of Sports Sciences, 26*(9), 935-940.

Karoly, P., Ruehlman, L. S., Okun, M. A., Lutz, R. S., Newton, C., & Fairholme, C. (2005). Perceived self-regulation of exercise goals and interfering goals among regular and irregular exercisers: A life space analysis. *Psychology of Sport and Exercise, 6*(4), 427-442.

Kelsey, K. S., DeVellis, B. M., Begum, M., Belton, L., Hooten, E. G., & Campbell, M. K. (2006). Positive affect, exercise and self-reported health in blue-collar women. *American Journal of Health Behavior, 30*(2), 199-207.

Kingi, D., Towers, A., Seebeck, R., & Flett, R. (2005). Pacific women's decisions about exercise adoption: Utilising the stage-of-exercise-adoption model. *The New Zealand Medical Journal, 118*(1216), U1493-U1493.

Kiviniemi, M. T., Voss-Humke, A. M., & Seifert, A. L. (2007). How do i feel about the behavior? the interplay of affective associations with behaviors and cognitive beliefs as influences on physical activity behavior. *Health Psychology, 26*(2), 152-158.

Kloek, G. C., van Lenthe, F. J., van Nierop, P. W. M., Schrijvers, C. T. M., & Mackenbach, J. P. (2006). Stages of change for moderate-intensity physical activity in deprived neighborhoods. *Preventive Medicine: An International Journal Devoted to Practice and Theory, 43*(4), 325-331.

Kolt, G.S., Oliver, M., Schofield, G.M., Kerse, N., Garrett, N., Latham, N.K. (2006). An overview and process evaluation of Telewalk: a telephone-based counseling intervention to encourage walking in older adults. *Health Promotion International, 21*(3), 201-208.

Lackner, C. (2005). Mediation from a group dynamics point of view. *Journal of Psychohistory, 33*(1), 41-46.

Landry, J. B., & Solmon, M. A. (2002). Self-determination theory as an organizing framework to investigate women's physical activity behavior. *Quest (00336297), 54*(4), 332-354.

LaRowe, T. L., Wubben, D. P., Cronin, K. A., Vannatter, S. M., & Adams, A. K. (2007). Development of a culturally appropriate, home-based nutrition and physical activity curriculum for Wisconsin American Indian families. *Preventing Chronic Disease, 4*(4), A109-A109.

Lee, Y., Park, N., & Kim, Y. (2006). Process of change, decisional balance, self-efficacy and depression across the stages of change for exercise among middle aged women in korea. *Taehan Kanho Hakhoe Chi, 36*(4), 587-595.

Levesque, L., Gauvin, L., & Desharnais, R. (2003). Maintaining exercise involvement: The role of learned resourcefulness in process of change use. *Psychology of Sport & Exercise, 4*(3), 237-253.

Lewis, B. A., Forsyth, L. H., Pinto, B. M., Bock, B. C., Roberts, M., & Marcus, B. H. (2006). Psychosocial mediators of physical activity in a randomized controlled intervention trial. *Journal of Sport & Exercise Psychology, 28*(2), 193-204.

Lippke, S., Ziegelmann, J. P., & Schwarzer, R. (2005). Stage-specific adoption and maintenance of physical activity: Testing a three-stage model. *Psychology of Sport and Exercise, 6*(5), 585-603.

Livaudais, J. C., Kaplan, C. P., Haas, J. S., Pérez-Stable, E. J., Stewart, S., & Des Jarlais, G. (2005). Lifestyle behavior counseling for women patients among a sample of California physicians. *Journal of Women's Health, 14*(6), 485-495.

Lorentzen, C., Ommundsen, Y., & Holme, I. (2007). Psychosocial correlates of stages of change in physical activity in an adult community sample. *European Journal of Sport Science, 7*(2), 93-106.

Lorentzen, C., Ommundsen, Y., Jenum, A.K. & Holme, I. (2007). The “Romsas in Motion” community intervention: program exposure and psychosocial mediated relationships to change in stages of change in physical activity. *International Journal of Behavioral Nutrition and Physical Activity, 4.*

Maerki, A., Bauer, G.B., Angst, E., Nigg, C.R., Gillmann, G., Gehring, T.M. (2006). Systematic counseling by general practitioners for promoting physical activity in elderly patients: a feasibility study. *Swiss Medical Weekly, 136*(29), 29-30.

Marquez, D.X. & McAuley, E. (2006). Social cognitive correlates of leisure time physical activity among Latinos. *Journal of Behavioral Medicine, 29*(3), 281-289.

Martin-Diener, E., Thüring, N., Melges, T., & Martin, B. W. (2004). The stages of change in three stage concepts and two modes of physical activity: A comparison of stage distributions and practical implications. *Health Education Research, 19*(4), 406-417.

Marttila, J., & Nupponen, R. (2003). Assessing stage of change for physical activity: How congruent are parallel methods? *Health Education Research, 18*(4), 419-428.

Matsudo, S. M., Matsudo, V. R., Andrade, D. R., Araújo, T. L., Andrade, E., de Oliveira, L., et al. (2004). Physical activity promotion: Experiences and evaluation of the agita são paulo program using the ecological mobile model. *Journal of Physical Activity & Health, 1*(2), 81-97.

McGannon, K. R., & Mauws, M. K. (2000). Discursive psychology: An alternative approach for studying adherence to exercise and physical activity. *Quest (00336297), 52*(2), 148-165.

Michie, S., Hardeman, W., Fanshawe, T., Prevost, A. T., Taylor, L., & Kinmonth, L. (2008). Investigating theoretical explanations for behaviour change: The case study of ProActive. *Psychology & Health, 23*(1), 25-39.

Nies, M. A., Artinian, N. T., Schim, S. M., Wal, J. S. V., & Sherrick-Escamilla, S. (2004). Effects of lay health educator interventions on activity, diet, and health risks in an urban Mexican American community. *Journal of Primary Prevention, 25*(4), 441-455.

Nies, M. A., & Kershaw, T. C. (2002). Psychosocial and environmental influences on physical activity and health outcomes in sedentary women. *Journal of Nursing Scholarship: An Official Publication of Sigma Theta Tau International Honor Society of Nursing / Sigma Theta Tau, 34*(3), 243-249.

Nies, M. A., Reisenberg, C. E., Chruscial, H. L., & Artibee, K. (2003). Southern women's response to a walking intervention. *Public Health Nursing (Boston, Mass.), 20*(2), 146-152.

O'Hea, E. L., Boudreaux, E. D., Jeffries, S. K., Carmack Taylor, C. L., Scarinci, I. C., & Brantley, P. J. (2004). Stage of change movement across three health behaviors: The role of self-efficacy. *American Journal of Health Promotion, 19*(2), 94-102.

Okun, M. A., Karoly, P., & Lutz, R. (2002). Clarifying the contribution of subjective norm to predicting leisure-time exercise. *American Journal of Health Behavior, 26*(4), 296-305.

Padula, C. A., & Burbank, P. M. (2002). Applying the transtheoretical model: Behavior change among family caregivers and nursing care staff. In P. M. Burbank, & D. Riebe (Eds.), *Promoting exercise and behavior change in older adults: Interventions with the transtheoretical model.* (pp. 269-298). New York, NY, US: Springer Publishing Co.

Plotnikoff, R. C., Hotz, S. B., Birkett, N. J., & Courneya, K. S. (2001). Exercise and the transtheoretical model: A longitudinal test of a population sample. *Preventive Medicine: An International Journal Devoted to Practice and Theory, 33*(5), 441-452.

Ransdell, L. B., Dratt, J., Kennedy, C., O'Neill, S., & DeVoe, D. (2001). Daughters and mothers exercising together (DAMET): A 12-week pilot project designed to improve physical self-perception and increase recreational physical activity. *Women & Health, 33*(3-4), 101-116.

Resnick, B. (2002). Testing the effect of the WALC intervention on exercise adherence in older adults. *Journal of Gerontological Nursing, 28*(6), 40-49.

Resnick, B., & Nigg, C. (2003). Testing a theoretical model of exercise behavior for older adults. *Nursing Research, 52*(2), 80-88.

Riebe, D., Blissmer, B., Greene, G., Caldwell, M., Ruggiero, L., Stillwell, K. M., et al. (2005). Long-term maintenance of exercise and healthy eating behaviors in overweight adults. *Preventive Medicine: An International Journal Devoted to Practice and Theory, 40*(6), 769-778.

Rodgers, W. M., Courneya, K. S., & Bayduza, A. L. (2001). Examination of the transtheoretical model and exercise in 3 populations. *American Journal of Health Behavior, 25*(1), 33-41.

Ronda, G., Van Assema, P., Candel, M., Ruland, E., Steenbakkers, M., Van Ree, J., et al. (2004). The dutch heart health community intervention 'hartslag limburg': Results of an effect study at individual level. *Health Promotion International, 19*(1), 21-31.

Rooney, B., Smalley, K., Larson, J., & Havens, S. (2003). Is knowing enough? increasing physical activity by wearing a pedometer. *WMJ: Official Publication of the State Medical Society of Wisconsin, 102*(4), 31-36.

Sargent, L. A. (2001). *The role of social support and self-efficacy in influencing moderate leisure time physical activity among African American women*. Eugene, OR; United States: Kinesiology Publications, University of Oregon.

Saunders, S. D., Garber, C. E., & Martins, D. (2002). Applying the transtheoretical model: Challenges with older adults from diverse ethnic and socioeconomic backgrounds. In P. M. Burbank, & D. Riebe (Eds.), *Promoting exercise and behavior change in older adults: Interventions with the transtheoretical model.* (pp. 235-267). New York, NY, US: Springer Publishing Co.

Schumann, A., Estabrooks, P.A., Nigg, C.R., & Hill, J. (2003). Validation of the stages of change with mild, moderate, and strenuous physical activity behavior, intentions, and self-efficacy. *International Journal of Sports medicine, 24*, 363-365.

Sears, S. R., & Stanton, A. L. (2001). Expectancy-value constructs and expectancy violation as predictors of exercise adherence in previously sedentary women. *Health Psychology: Official Journal of the Division of Health Psychology, American Psychological Association, 20*(5), 326-333.

Segar, M., Jayaratne, T., Hanlon, J., & Richardson, C. R. (2002). Fitting fitness into women's lives: Effects of a gender-tailored physical activity intervention. *Women's Health Issues: Official Publication of the Jacobs Institute of Women's Health, 12*(6), 338-347.

Shields, C. A., Brawley, L. R., & Lindover, T. I. (2006). Self-efficacy as a mediator of the relationship between causal attributions and exercise behavior. *Journal of Applied Social Psychology, 36*(11), 2785-2802.

Smith, R. A., & Biddle, S. J. H. (1999). Attitudes and exercise adherence: Test of the theories of reasoned action and planned behaviour. *Journal of Sports Sciences, 17*(4), 269-281.

Stewart, A. L. (2001). Community-based physical activity programs for adults age 50 and older. *Journal of Aging & Physical Activity, 9*, S71-s91.

Taylor-Piliae, R. E., Haskell, W. L., Waters, C. M., & Froelicher, E. S. (2006). Change in perceived psychosocial status following a 12-week tai chi exercise programme. *Journal of Advanced Nursing, 54*(3), 313-329.

Tessaro, I. A., Taylor, S., Belton, L., Campbell, M. K., Benedict, S., Kelsey, K., et al. (2000). Adapting a natural (lay) helpers model of change for worksite health promotion for women. *Health Education Research, 15*(5), 603-614.

Thornton, P. L., Kieffer, E. C., Salabarría-Peña, Y., Odoms-Young, A., Willis, S. K., Kim, H., et al. (2006). Weight, diet, and physical activity-related beliefs and practices among pregnant and postpartum latino women: The role of social support. *Maternal & Child Health Journal, 10*(1), 95-104.

Thrane, C. (2006). Explaining educational-related inequalities in health: Mediation and moderator models. *Social Science & Medicine (1982), 62*(2), 467-478.

Thrasher, J. F., Campbell, M. K., & Oates, V. (2004). Behavior-specific social support for healthy behaviors among African American church members: Applying optimal matching theory. *Health Education & Behavior, 31*(2), 193-205.

Tucker, P., & Irwin, J. D. (2006). Feasibility of a campus-based "buddy system" to promote physical activity: Canadian students' perspectives. *Journal of Physical Activity & Health, 3*(3), 323-334.

Tudor-Locke, C., & Chan, C. B. (2006). An exploratory analysis of adherence patterns and program completion of a pedometer-based physical activity intervention. *Journal of Physical Activity & Health, 3*(2), 210-220.

Tufano, J.T. & Karras, B.T. (2005). Mobile eHealth interventions for obesity: a timely opportunity to leverage convergence trends. *Journal of medical Internet Research, 7*(5), 97-104.

Umstattd, M. R., & Hallam, J. (2007). Older adults' exercise behavior: Roles of selected constructs of social-cognitive theory. *Journal of Aging and Physical Activity, 15*(2), 206-218.

Umstattd, M. R., & Hallam, J. S. (2006). Use of social cognitive theory variables across exercise stages of change of employed women. *American Journal of Health Studies, 21*(1), 44-48.

Umstattd, M. R., Saunders, R., Wilcox, S., Valois, R. F., & Dowda, M. (2006). Correlates of self-regulation for physical activity among older adults. *American Journal of Health Behavior, 30*(6), 710-719.

Wallace, L. S., & Buckworth, J. (2003). Longitudinal shifts in exercise stages of change in college students. *The Journal of Sports Medicine and Physical Fitness, 43*(2), 209-212.

Westerståhl, A., Segesten, K., & Björkelund, C. (2002). Integration of information about cardiovascular risk factors: How do highly motivated women in a lifestyle intervention programme act and react? *Scandinavian Journal of Primary Health Care, 20*(1), 22-27.

Whaley, D. E. (2003). Future-oriented self-perceptions and exercise behavior in middle-aged women. *Journal of Aging and Physical Activity, 11*(1), 1-17.

Wilbur, J., Vassalo, A., Chandler, P., McDevitt, J., & Miller, A. M. (2005). Midlife women's adherence to home-based walking during maintenance. *Nursing Research, 54*(1), 33-40.

Wilcox, S., Castro, C. M., & King, A. C. (2006). Outcome expectations and physical activity participation in two samples of older women. *Journal of Health Psychology, 11*(1), 65-77.

Williams, D. M., Matthews, C. E., Rutt, C., Napolitano, M. A., & Marcus, B. H. (2008). Interventions to increase walking behavior. *Medicine & Science in Sports & Exercise, 40*, S567-S573.

Winett, R.A., Anderson, E.S., et al. (1999). Church-based health behavior programs: using social cognitive theory to formulate interventions for at-risk populations. *Applied & Preventive Psychology, 8*(2), 129-142.

Witmer, J. M., Hensel, M. R., Holck, P. S., Ammerman, A. S., & Will, J. C. (2004). Heart disease prevention for Alaska native women: A review of pilot study findings. *Journal of Women's Health (2002), 13*(5), 569-578.

No Mediating Variables examined

Abbema, E.A., Assema van, P., Leeuw de, E., & Vries de, N.K. (2004). Effect evaluation of a comprehensive community intervention aimed at reducing socioeconomic health inequalities in the Netherlands. *Health Promotion International, 19*, 141-156.

Ackermann, R. T., Deyo, R. A., & LoGerfo, J. P. (2005). Prompting primary providers to increase community exercise referrals for older adults: A randomized trial. *Journal of the American Geriatrics Society, 53*(2), 283-289.

Albright, C.L., Pruitt, L., Castro, C., Gonzales, A., Woo, S., & King, A.C. (2005). Modifying physical activity in a multiethnic sample of low-income women: One-year results from the IMPACT project. *Annals of Behavior medicine, 30*(3), 191-200.

Aldana, S. G., Greenlaw, R. L., Diehl, H. A., Salberg, A., Merrill, R. M., & Ohmine, S. (2005). The effects of a worksite chronic disease prevention program. *Journal of Occupational and Environmental Medicine / American College of Occupational and Environmental Medicine, 47*(6), 558-564.

Almeida, F. A., Smith-Ray, R. L., van den Berg, R., Schriener, P., Gonzales, M., Onda, P., et al. (2005). Utilizing a simple stimulus control strategy to increase physician referrals for physical activity promotion. *Journal of Sport & Exercise Psychology, 27*(4), 505.

Amati, F., Barthassat, V., Miganne, G., Hausman, I., Monnin, D. G., Costanza, M. C., et al. (2007). Enhancing regular physical activity and relapse prevention through a 1-day therapeutic patient education workshop: A pilot study. *Patient Education and Counseling, 68*(1), 70-78.

Annesi, J. J., & Unruh, J. L. (2007). Effects of the COACH APPROACH intervention on drop-out rates among adults initiating exercise programs at nine YMCAs over three years. *Perceptual and Motor Skills, 104*(2), 459-466.

Bertozzi, N., Bakken, E., Bolognesi, M., Castoldi, F., Massarini, M., Galvani, C., et al. (2004). Promoting physical activity in overweight and obese patients: Counseling in primary care from Italy (cesena, 2002–2003). *Sport Sciences for Health, 1*(1), 25-30.

Blissmer, B., & McAuley, E. (2002). Testing the requirements of stages of physical activity among adults: The comparative effectiveness of stage-matched, mismatched, standard care, and control interventions. *Annals of Behavioral Medicine: A Publication of the Society of Behavioral Medicine, 24*(3), 181-189.

Brawley, L. R., Rejeski, W. J., & Lutes, L. (2000). A group-mediated cognitive-behavioral intervention for increasing adherence to physical activity in older adults. *Journal of Applied Biobehavioral Research, 5*(1), 47-65.

Bray, S. R., Clayton, C. S., Kwan, M. Y. W., Arbour, K. P., & Chohan, J. (2007). Effects of a first-year student physical activity guide on action plans and physical activity during transition to university. *Journal of Sport & Exercise Psychology, 29*, S148-S149.

Campbell, M. K., Tessaro, I., DeVellis, B., Benedict, S., Kelsey, K., Belton, L., et al. (2002). Effects of a tailored health promotion program for female blue-collar workers: Health works for women. *Preventive Medicine: An International Journal Devoted to Practice and Theory, 34*(3), 313-323.

Cardinal, B. J., Jacques, K. M., & Levy, S. S. (2002). Evaluation of a university course aimed at promoting exercise behavior. / evaluation d ' un cours universitaire pour la promotion de l ' exercice. *Journal of Sports Medicine & Physical Fitness, 42*(1), 113-119.

Cheung, C., Wyman, J., Gross, C., Peters, J., Findorff, M., & Stock, H. (2007). Exercise behavior in older adults: A test of the transtheoretical model. *Journal of Aging and Physical Activity, 15*(1), 103-118.

Clark, D. O., Stump, T. E., & Damush, T. M. (2003). Outcomes of an exercise program for older women recruited through primary care. *Journal of Aging and Health, 15*(3), 567-585.

Cole, G., Leonard, B., Hammond, S., & Fridinger, F. (1998). Using "stages of behavioral change" constructs to measure the short-term effects of a worksite-based intervention to increase moderate physical activity. *Psychological Reports, 82*(2), 615-618.

Conn, V. S., Burks, K. J., Minor, M. A., & Mehr, D. R. (2003). Randomized trial of 2 interventions to increase older women's exercise. *American Journal of Health Behavior, 27*(4), 380-388.

De Cocker, K. A., De Bourdeaudhuij, I. M., Brown, W. J., & Cardon, G. M. (2007). Effects of '10,000 steps ghent': A whole-community intervention. *American Journal of Preventive Medicine, 33*(6), 455-463.

De Cocker, K.A., De Bourdeaudhuij, I.M., & Cardon, G.M. (2008). The effect of pedometer use in combination with cognitive and behavioral support materials to promote physical activity. *Patient Education and counseling, 70*, 209-214.

de Vries, H., Kremers, S. P. J., Smeets, T., Brug, J., & Eijmael, K. (2008). The effectiveness of tailored feedback and action plans in an intervention addressing multiple health behaviors. *American Journal of Health Promotion, 22*(6), 417-425.

DiBrezzo, R., Shadden, B.B., Raybon, B.H., Powers, M. (2005). Exercise intervention designed to improve strength and dynamic balance among community dwelling older adults. *Journal of Aging and Physical Activity, 13*(2), 198-209.

Dornelas, E. A., Stepnowski, R. R., Fischer, E. H., & Thompson, P. D. (2007). Urban ethnic minority women's attendance at health clinic vs. church based exercise programs. *Journal of Cross-Cultural Gerontology, 22*(1), 129-136.

Dutton, G. R., Martin, P. D., Welsch, M. A., & Brantley, P. J. (2007). Promoting physical activity for low-income minority women in primary care. *American Journal of Health Behavior, 31*(6), 622-631.

DuVall, C., Dinger, M. K., Taylor, E. L., & Bemben, D. (2004). Minimal-contact physical activity interventions in women: A pilot study. *American Journal of Health Behavior, 28*(3), 280-286.

Dzator, J.A., Hendrie, D., Burke, V., Gianguilio, N., Gillam, H.F., Beilin, L.J., & Houghton, S. (2004). A randomized trial of interactive group sessions achieved greater improvements in nutrition and physical activity at a tiny increase in cost. *Journal of Clinical Epidemiology, 57*, 610-619.

Elliot, D. L., Goldberg, L., Kuehl, K. S., Moe, E. L., Breger, R. K. R., & Pickering, M. A. (2007). The PHLAME (promoting healthy lifestyles: Alternative models' effects) firefighter study: Outcomes of two models of behavior change. *Journal of Occupational & Environmental Medicine, 49*(2), 204-213.

Emmons, K. M., Stoddard, A. M., Fletcher, R., Gutheil, C., Suarez, E. G., Lobb, R., et al. (2005). Cancer prevention among working class, multiethnic adults: Results of the healthy directions--health centers study. *American Journal of Public Health, 95*(7), 1200-1205.

Gaston, M. H., Porter, G. K., & Thomas, V. G. (2007). Prime time sister circles: Evaluating a gender-specific, culturally relevant health intervention to decrease major risk factors in mid-life african-american women. *Journal of the National Medical Association, 99*(4), 428-438.

Green, B.B., McAfee, T., Hindmarsh, M., Madsen, L., Caplow, M., & Buist, D. (2002). Effectiveness of telephone support in increasing physical activity levels in primary care patients. *American Journal of preventive medicine, 22*, 177-183.

Hall, P. A., & Fong, G. T. (2003). The effects of a brief time perspective intervention for increasing physical activity among young adults. *Psychology & Health, 18*(6), 685-706.

Heschka S., Anderson, J.W., Atkinson, R.L., Greenway, F.L., et al. (2003). Weight loss with self-help compared with a structured commercial program: a randomized trial. *JAMA, 289*, 1792-1798.

Irwin, M.L., Tworoger, S.S., et al. (2004). Influence of demographic, physiologic, and psychological variables on adherence to a yearlong moderate-intensity exercise trial in postmenopausal women. *Preventive Medicine, 39*(6), 1080-1086.

Jackicic, J.M., Marcus, B.H., Gallagher, K.I., Napolitano, M., & Lang, W. (2003). Effect of exercise duration and intensity on weight loss in overweight, sedentary women. *Journal of American Medical Association, 290*, 1323-1330.

Jancey, J. M., Clarke, A., Howat, P. A., Lee, A. H., Shilton, T., & Fisher, J. (2008). A physical activity program to mobilize older people: A practical and sustainable approach. *The Gerontologist, 48*(2), 251-257.

Jenum, A. K., Anderssen, S. A., Birkeland, K. I., Holme, I., Graff-Iversen, S., Lorentzen, C., et al. (2006). Promoting physical activity in a low-income multiethnic district: Effects of a community intervention study to reduce risk factors for type 2 diabetes and cardiovascular disease: A community intervention reducing inactivity. *Diabetes Care, 29*(7), 1605-1612.

Jimmy, G., & Martin, B. W. (2005). Implementation and effectiveness of a primary care based physical activity counselling scheme. *Patient Education and Counseling, 56*(3), 323-331.

Keele-Smith, R., Leon, T. (2003). Evaluation of individually tailored interventions on exercise adherence. *Western Journal of Nursing, 25*(6), 623-640.

Kramish Campbell, M., James, A., Hudson, M. A., Carr, C., Jackson, E., Oakes, V., et al. (2004). Improving multiple behaviors for colorectal cancer prevention among African American church members. *Health Psychology, 23*(5), 492-502.

Kypri, K., & McAnally, H. M. (2005). Randomized controlled trial of a web-based primary care intervention for multiple health risk behaviors. *Preventive Medicine: An International Journal Devoted to Practice and Theory, 41*(3), 761-766.

Lingfors, H., Lindström, K., Persson, L., Bengtsson, C., & Lissner, L. (2003). Lifestyle changes after a health dialogue: Results from the live for life health promotion program. *Scandinavian Journal of Primary Health Care, 21*(4), 248-252.

Logue, E., Sutton, K., Jarjoura, D., Smucker, W., Baughman, K., & Capers, C. (2005). Transtheoretical model-chronic disease care for obesity in primary care: A randomized trial. *Obesity Research, 13*(5), 917-927.

Martin, M. Y., Person, S. D., Kratt, P., Prayor-Patterson, H., Kim, Y., Salas, M., et al. (2008). Relationship of health behavior theories with self-efficacy among insufficiently active hypertensive African-American women. *Patient Education and Counseling, 72*(1), 137-145.

Mau, M. K., Glanz, K., Severino, R., Grove, J. S., Johnson, B., & Curb, J. D. (2001). Mediators of lifestyle behavior change in native Hawaiians: Initial findings from the native Hawaiian diabetes intervention program. *Diabetes Care, 24*(10), 1770-1775.

Morey, M. C., Dubbert, P. M., Doyle, M. E., MacAller, H., Crowley, G. M., Kuchibhatla, M., et al. (2003). From supervised to unsupervised exercise: Factors associated with exercise adherence. *Journal of Aging and Physical Activity, 11*(3), 351-368.

Mutrie, N., Carney, C., Blamey, A., Crawford, F., Aitchison, T., & Whitelaw, A. (2002). "Walk in to work out": A randomised controlled trial of a self help intervention to promote active commuting. *Journal of Epidemiology and Community Health, 56*(6), 407-412.

Napolitano, M. A., Fotheringham, M., Tate, D., Sciamanna, C., Leslie, E., Owen, N., et al. (2003). Evaluation of an internet-based physical activity intervention: A preliminary investigation. *Annals of Behavioral Medicine: A Publication of the Society of Behavioral Medicine, 25*(2), 92-99.

Napolitano, M. A., Whiteley, J. A., Papandonatos, G., Dutton, G., Farrell, N. C., Albrecht, A., et al. (2006). Outcomes from the women's wellness project: A community-focused physical activity trial for women. *Preventive Medicine, 43*(6), 447-453.

Oden, M. P. (2005). *The relationship between awareness of cardiac risk factors, physical activity level, telephone intervention, and health behavior change in African-American women*. US: ProQuest Information & Learning.

Orti, E. S., & Donaghy, M. (2004). A cognitive-behavioural intervention to increase adherence of adult women exercisers. *Advances in Physiotherapy, 6*(2), 84-92.

Petrella, R. J., Koval, J. J., Cunningham, D. A., & Paterson, D. H. (2003). Can primary care doctors prescribe exercise to improve fitness? the step test exercise prescription (STEP) project. *American Journal of Preventive Medicine, 24*(4), 316-322.

Pinto, B., Friedman, R., Marcus, B. H., Kelley, H., Tennstedt, S., & Gillman, M. W. (2002). Effects of a computer-based, telephone-counseling system on physical activity. *American Journal of Preventive Medicine, 23*(2), 113-120.

Plotnikoff, R. C., Brunet, S., Courneya, K. S., Spence, J. C., Birkett, N. J., Marcus, B., et al. (2007). The efficacy of stage-matched and standard public health materials for promoting physical activity in the workplace: The physical activity workplace study (PAWS). *American Journal of Health Promotion, 21*(6), 501-509.

Poston, W. S. C.,II, Haddock, K., Olvera, N. E., Suminski, R. R., Reeves, R. S., Dunn, J. K., et al. (2001). Evaluation of a culturally appropriate intervention to increase physical activity. *American Journal of Health Behavior, 25*(4), 396-406.

Proper, K. I., van der Beek, A. J., Hildebrandt, V. H., Twisk, J. W. R., & van Mechelen, W. (2003). Short term effect of feedback on fitness and health measurements on self reported appraisal of the stage of change. / effet a court terme d ' un feedback sur les mesures de l ' etat de forme et de sante sur l ' evaluation subjective de sa situation dans une demarche de changement. *British Journal of Sports Medicine, 37*(6), 529-534.

Proper, K.I., Hilderbrandt, V.H., Van der Beek, A.J., Twisk, J. W. R., van Mechelen, W. (2003). Effect of individual counseling on physical activity fitness and health. *American Journal of Preventive Medicine, 24*(3), 218-225.

Purath, J., Miller, A. M., McCabe, G., & Wilbur, J. (2004). A brief intervention to increase physical activity in sedentary working women. *CJNR: Canadian Journal of Nursing Research, 36*(1), 76-91.

Resnicow, K., Jackson, A., Blissett, D., Wang, T., et al. (2005). Results of the healthy body healthy spirit trial. *Health Psychology, 24*, 339-348.

Richeson, N. E., Croteau, K. A., Jones, D. B., & Farmer, B. C. (2006). Effects of a pedometer-based intervention on the physical performance and mobility-related self-efficacy of community-dwelling older adults: An interdisciplinary preventive health care intervention. *Therapeutic Recreation Journal, 40*(1), 18-32.

Sallis, J. F., Calfas, K. J., Nichols, J. F., Sarkin, J. A., Johnson, M. F., Caparosa, S., et al. (1999). Evaluation of a university course to promote physical activity: Project GRAD. / evaluation d'un cours universitaire destine a promouvoir l'activite physique : Projet GRAD. *Research Quarterly for Exercise & Sport, 70*(1), 1-10.

Sallis, J. F., Patrick, K., Calfas, K. J., Zabinski, M. F., Prochaska, J. J., Thompson, S., et al. (1999). A multi-media behavior change program for nutrition and physical activity in primary care: PACE+ for adults. *Homeostasis in Health and Disease, 39*(5), 196-202.

Shirazi, K. K., Wallace, L. M., Niknami, S., Hidarnia, A., Torkaman, G., Gilchrist, M., et al. (2007). A home-based, transtheoretical change model designed strength training intervention to increase exercise to prevent osteoporosis in Iranian women aged 40-65 years: A randomized controlled trial. *Health Education Research, 22*(3), 305-317.

Simons-Morton, D. G., Blair, S. N., King, A. C., Morgan, T. M., Applegate, W. B., O'Toole, M., et al. (2001). Effects of physical activity counseling in primary care: The activity counseling trial: A randomized controlled trial. *JAMA: Journal of the American Medical Association, 286*(6), 677-687.

Smeets, T., Kremers, S. P. J., de Vries, H., & Brug, J. (2007). Effects of tailored feedback on multiple health behaviors. *Annals of Behavioral Medicine, 33*(2), 117-123.

Sorensen, G., Barbeau, E., Hunt, M. K., Kaphingst, K., Wallace, L., & Stoddard, A. M. (2005). Promoting behavior change among working-class, multiethnic workers: Results of the healthy directions-small business study. *American Journal of Public Health, 95*(8), 1389-1395.

Steele, R., Mummery, W. K., & Dwyer, T. (2007). Using the internet to promote physical activity: A randomized trial of intervention delivery modes. *Journal of Physical Activity & Health, 4*(3), 245-260.

Stovitz, S. D., VanWormer, J. J., Center, B. A., & Bremer, K. L. (2005). Pedometers as a means to increase ambulatory activity for patients seen at a family medicine clinic. *The Journal of the American Board of Family Practice / American Board of Family Practice, 18*(5), 335-343.

Sugden, J. A., Sniehotta, F. F., Donnan, P. T., Boyle, P., Johnston, D. W., & McMurdo, M. E. T. (2008). The feasibility of using pedometers and brief advice to increase activity in sedentary older women--a pilot study. *BMC Health Services Research, 8*, 169-169.

Tan, E. J., Xue, Q., Li, T., Carlson, M. C., & Fried, L. P. (2006). Volunteering: A physical activity intervention for older adults--the experience corps program in Baltimore. *Journal of Urban Health: Bulletin of the New York Academy of Medicine, 83*(5), 954-969.

Vandelanotte, C., De Bourdeaudhuij, I., & Brug, J. (2007). Two-year follow-up of sequential and simultaneous interactive computer-tailored interventions for increasing physical activity and decreasing fat intake. *Annals of Behavioral Medicine, 33*(2), 213-219.

Wellman, N. S., Kamp, B., Kirk-Sanchez, N. J., & Johnson, P. M. (2007). Eat better & move more: A community-based program designed to improve diets and increase physical activity among older Americans. *American Journal of Public Health, 97*(4), 710-717.

Whitehead, D., Bodenlos, J.S., Cowles, M.L., Jones, G.N., & Brantley, P.J. (2007). A Stage-targeted physical activity intervention among a predominantly African-American low-income primary care population. *American Journal of Health Promotion, 21*(3), 160-163.

Wilbur, J., McDevitt, J. H., Wang, E., Dancy, B. L., Miller, A. M., Briller, J., et al. (2008). Outcomes of a home-based walking program for African-American women. *American Journal of Health Promotion, 22*(5), 307-317.

Wilcox, S., Dowda, M., Leviton, L. C., Bartlett-Prescott, J., Bazzarre, T., Campbell-Voytal, K., et al. (2008). Active for life final results from the translation of two physical activity programs. *American Journal of Preventive Medicine, 35*(4), 340-351.

Winett, R. A., Anderson, E. S., Wojcik, J. R., Winett, S. G., & Bowden, T. (2007). Guide to health: Nutrition and physical activity outcomes of a group-randomized trial of an internet-based intervention in churches. *Annals of Behavioral Medicine: A Publication of the Society of Behavioral Medicine, 33*(3), 251-261.

Wylie-Rosett, J., Swencionis, C., Ginsberg, M., Cimino, C., Wassertheil-Smoller, S., Caban, A., et al. (2001). Computerized weight loss intervention optimizes staff time: The clinical and cost results of a controlled clinical trial conducted in a managed care setting. *Journal of the American Dietetic Association, 101*(10), 1155.

Yancey, A. K., McCarthy, W. J., Taylor, W. C., Merlo, A., Gewa, C., Weber, M. D., et al. (2004). The Los Angeles lift off: A sociocultural environmental change intervention to integrate physical activity into the workplace. *Preventive Medicine: An International Journal Devoted to Practice and Theory, 38*(6), 848-856.

Yanek, L. R., Becker, D. M., Moy, T. F., Gittelsohn, J., & Koffman, D. M. (2001). Project joy: Faith based cardiovascular health promotion for African American women. *Public Health Reports (Washington, D.C.: 1974), 116 Suppl 1*, 68-81.

No PA measure

Anderson, R. T., King, A., Stewart, A. L., Camacho, F., & Rejeski, W. J. (2005). Physical activity counseling in primary care and patient well-being: Do patients benefit? *Annals of Behavioral Medicine: A Publication of the Society of Behavioral Medicine, 30*(2), 146-154.

Annesi, J. J. (2007). Relations of changes in exercise self-efficacy, physical self-concept, and body satisfaction with weight changes in obese white and African American women initiating a physical activity program. *Ethnicity & Disease, 17*(1), 19-22.

Brassington, G. S., Atienza, A., Perczek, R. E., DiLorenzo, T. M., & King, A. C. (2002). Intervention-related cognitive versus social mediators of exercise adherence in the elderly. *American Journal of Preventive Medicine, 23*(2), 80-86.

Burke, V., Giangiulio, N., Gillam, H. F., Beilin, L. J., & Houghton, S. (2004). Changes in cognitive measures in a randomized controlled trial of a health promotion program for couples targeting diet and physical activity. *American Journal of Health Promotion: AJHP, 18*(4), 300-311.

Caperchione, C., & Mummery, K. (2007). Psychosocial mediators of group cohesion on physical activity intention of older adults. *Psychology, Health & Medicine, 12*(1), 81-93.

Carlson, L. E., Taenzer, P., Koopmans, J., & Casebeer, A. (2003). Predictive value of aspects of the transtheoretical model on smoking cessation in a community-based, large-group cognitive behavioral program. *Addictive Behaviors, 28*(4), 725-740.

Castro, C. M., King, A. C., & Brassington, G. S. (2001). Telephone versus mail interventions for maintenance of physical activity in older adults. *Health Psychology, 20*(6), 438-444.

Chatzisarantis, N.L.D., Hagger, M.S., Wang, J.C.K. (2008). An experimental test of cognitive dissonance theory in the domain of physical exercise. *Journal of Applied Sport Psychology, 20*(1), 97-115.

Clarke, K. K., Freeland-Graves, J., Klohe-Lehman, D. M., Milani, T. J., Nuss, H. J., & Laffrey, S. (2007). Promotion of physical activity in low-income mothers using pedometers. *Journal of the American Dietetic Association, 107*(6), 962-967.

Cox, K. L., Gorely, T. J., Puddey, I. B., Burke, V., & Beilin, L. J. (2003). Exercise behaviour change in 40 to 65-year-old women: The SWEAT study (sedentary women exercise adherence trial). *British Journal of Health Psychology, 8*, 477-495.

Findorff, M. J., Stock, H. H., Gross, C. R., & Wyman, J. F. (2007). Does the transtheoretical model (TTM) explain exercise behavior in a community-based sample of older women? *Journal of Aging and Health, 19*(6), 985-1003.

Goldstein, M. G., Pinto, B. M., Marcus, B. H., Lynn, H., Jette, A. M., McDermott, S., et al. (1999). Physician-based physical activity counseling for middle-aged and older adults: A randomized trial. *Annals of Behavioral Medicine, 21*(1), 40-47.

Gorely, T., & Bruce, D. (2000). A 6-month investigation of exercise adoption from the contemplation stage of the transtheoretical model. *Psychology of Sport and Exercise, 1*(2), 89-101.

Griffin-Blake, C. S., & DeJoy, D. M. (2006). Evaluation of social-cognitive versus stage-matched, self-help physical activity interventions at the workplace. *American Journal of Health Promotion, 20*(3), 200-209.

Hurling, R., Fairley, B. W., & Dias, M. B. (2006). Internet-based exercise intervention systems: Are more interactive designs better? *Psychology & Health, 21*(6), 757-772.

Jones, N.D., DellaCorte, M.R., et al. (2001). Seniorcise: A print exercise intervention for older adults. *Educational Gerontology, 27*(8), 717-728.

Jones, L.W., Sinclair, R.C., & Courneya, K.S. (2003). The effects of source and message framing on exercise intentions, behaviors, and attitudes: an integration of the elaboration likelihood model and prospect theory. *Journal of Applied Social Psychology, 33*, 179-196.

Kao, Y., Lu, C., & Huang, Y. (2002). Impact of a transtheoretical model on the psychosocial factors affecting exercise among workers. *The Journal of Nursing Research: JNR, 10*(4), 303-310.

Kelley, K. & Abraham, C. (2004). RCT of a theory-based intervention promoting healthy eating and physical activity amongst out-patients older than 65 years. *Social Science & Medicine, 59*(4), 787-797.

Kerr, N. L., Messé, L. A., Seok, D., Sambolec, E. J., Lount, R. B., & Park, E. S. (2007). Psychological mechanisms underlying the köhler motivation gain. *Personality and Social Psychology Bulletin, 33*(6), 828-841.

Li, F., Harmer, P., McAuley, E., Fisher, K. J., Duncan, T. E., & Duncan, S. C. (2001). Tai chi, self-efficacy, and physical function in the elderly. *Prevention Science: The Official Journal of the Society for Prevention Research, 2*(4), 229-239.

Li, F., McAuley, E., Harmer, P., Duncan, T. E., & Chaumeton, N. R. (2001). Tai chi enhances self-efficacy and exercise behavior in older adults. *Journal of Aging & Physical Activity, 9*(2), 161-171.

Martinson, B. C., Crain, A. L., Sherwood, N. E., Hayes, M., Pronk, N. P., & O'Connor, P. J. (2008). Maintaining physical activity among older adults: Six-month outcomes of the keep active Minnesota randomized controlled trial. *Preventive Medicine, 46*(2), 111-119.

McQuigg, M., Brown, J., Broom, J., Laws, R. A., Reckless, J. P. D., Noble, P. A., et al. (2005). Empowering primary care to tackle the obesity epidemic: The counterweight programme. *European Journal of Clinical Nutrition, 59 Suppl 1*, S93.

Mihalko, S.L., Wickley, K.L., & Sharpe, B. L. (2006). Promoting physical activity in independent living communities. *Medicine and Science in Sports and Exercise, 38*(1), 112-115.

Naylor, P. J., Simmonds, G., Riddoch, C., Velleman, G., & Turton, P. (1999). Comparison of stage-matched and unmatched interventions to promote exercise behaviour in the primary care setting. *Health Education Research, 14*(5), 653-666.

Perry, C. K., Rosenfeld, A. G., Bennett, J. A., & Potempa, K. (2007). Heart-to-heart: Promoting walking in rural women through motivational interviewing and group support. *The Journal of Cardiovascular Nursing, 22*(4), 304-312.

Pfeffer, I., & Alfermann, D. (2008). Initiation of physical exercise: An intervention study based on the transtheoretical model. *International Journal of Sport Psychology, 39*(1), 41-58.

Prochaska, J. O., Butterworth, S., Redding, C. A., Burden, V., Perrin, N., Leo, M., et al. (2008). Initial efficacy of MI, TTM tailoring and HRI's with multiple behaviors for employee health promotion. *Preventive Medicine, 46*(3), 226-231.

Purath, J., & Miller, A. M. (2005). Predictors of improvement in women's physical activity. *Women & Health, 42*(3), 57-75.

Rhodes, R. E., Martin, A. D., & Taunton, J. E. (2001). Temporal relationships of self-efficacy and social support as predictors of adherence in a 6-month strength-training program for older women. *Perceptual and Motor Skills, 93*(3), 693-703.

Rhodes, R. E., & Courneya, K. S. (2005). Threshold assessment of attitude, subjective norm, and perceived behavioral control for predicting exercise intention and behavior. *Psychology of Sport and Exercise, 6*(3), 349-361.

Rovniak, L. S. (2007). *Operationalizing mastery experiences in e-mail-based fitness walking programs*. US: ProQuest Information & Learning.

Stevens, M., Lemmink, K. A. P. M., Greef, M. H. G. d., & Rispens, P. (2000). Groningen active living model (GALM): Stimulating physical activity in sedentary older adults; first results. *Preventive Medicine: An International Journal Devoted to Practice and Theory, 31*(5), 547-553.

Stevens, M., Lemmink, K. A. P. M., van Heuvelen, M. J. G., de Jong, J., & Rispens, P. (2003). Groningen active living model (GALM): Stimulating physical activity in sedentary older adults; validation of the behavioral change models. *Preventive Medicine: An International Journal Devoted to Practice and Theory, 37*(6), 561-570.

Suminski, R.R., Petosa, R. (2006). Web-assisted instruction for changing social cognitive variables related to physical activity. *Journal of American College Health, 54*(4), 219-225.

Toft, U. N., Kristoffersen, L. H., Aadahl, M., von Huth Smith, L., Pisinger, C., & Jorgensen, T. (2007). Diet and exercise intervention in a general population: Mediators of participation and adherence: The Inter99 study. *European Journal of Public Health, 17*(5), 455-463.

Veverka, D.V., Anderson, J., Auld, G.W., Coulter, G.R., Kennedy, C., Chapman, P.L. (2003). Use of the stages of change model in improving nutrition and exercise habits in enlisted air force men. *Military Medicine, 168,* 373-379.

White, J. L., & Ransdell, L. B. (2003). Worksite intervention model for facilitating changes in physical activity, fitness, and psychological parameters. *Perceptual & Motor Skills, 97*(2), 461-466.

Williams, D. M., Papandonatos, G. D., Napolitano, M. A., Lewis, B. A., Whiteley, J. A., & Marcus, B. H. (2006). Perceived enjoyment moderates the efficacy of an individually tailored physical activity intervention. *Journal of Sport & Exercise Psychology, 28*(3), 300-309.

Woods, C., Mutrie, N., & Scott, M. (2002). Physical activity intervention: A transtheoretical model-based intervention designed to help sedentary young adults become active. *Health Education Research, 17*(4), 451-460.

Dissertations, Thesis, Abstracts, Meeting/conference abstracts

Berlant, N. E. (2004). *Increasing adherence to an exercise intervention*. US: ProQuest Information & Learning.

Blissmer, B., & McAuley, E. (2002). Predicting quality of life outcomes from a lifestyle activity intervention: Impact of self-efficacy and activity level. (abstract). *Journal of Sport & Exercise Psychology, 24*, S35-S35.

Byfield, C. L. (2001). *Development and evaluation of a lifestyle physical activity intervention for obese sedentary women*. US: ProQuest Information & Learning.

Dean, M. L. (2001). *Effects of vow-making on adherence to a 12-week personal fitness program, self-efficacy, and theory of planned behavior constructs*. Eugene, OR; United States: Kinesiology Publications, University of Oregon.

Fahrenwald, N. L. (2003). *'Moms on the move': A physical activity intervention for WIC mothers*. US: ProQuest Information & Learning.

Feathers, J.T., Kieffer, E., Anderson, M., Guzman, G., James, S. (2003). REACH 2010: evaluation of a healthy lifestyle intervention for African Americans and Latinos in Detroit. *Diabetes, 52*, A492.

Foy, C. G. (1999). *An examination of social support as a facilitator of exercise adherence among African-American women*. US: ProQuest Information & Learning.

Gill, D. S. (1999). *Testing a biopsychosocial model of health behavior: A community intervention for reducing obesity in Mexican-American women*. US: ProQuest Information & Learning.

Harder, M. (1999). *The effects of stage-matched intervention on the stages of change and exercise self-efficacy*. Eugene, Or.; United States: Microform Publications, University of Oregon.

Hardy, A. M. (2000). *Evaluation of an internet, stage-based physical activity intervention*. Eugene, OR; United States: Microform Publications, University of Oregon.

Havenar, J. (2007). *Adapted motivational interviewing for increasing physical activity: A 12 month clinical trial*. US: ProQuest Information & Learning.

Hsiao, E. T. Y. (2003). *Using message framing to promote regular physical activity in college-age women and men*. US: ProQuest Information & Learning.

Hurley, K. S. (2001). *The effectiveness of a goal setting intervention in enhancing women's self-efficacy to overcome exercise barriers*. Eugene, OR; United States: Kinesiology Publications, University of Oregon.

Kitzman, H.E., Dunn, A.L., Barlow, C.E., Kampert, J.B. (2001). Project PRIME: family and peer support predicts maintenance at 24 months. *Medicine and Science in Sports and Exercise, 33*(5), S101.

Laplante, M. (2004). *The effects of stage-matched, stage-mismatched, and standard care interventions on physical activity behavior, stage transition, and hypothesized mediators of change: Test of a stage model*. US: ProQuest Information & Learning.

Levy, S. S. (2002). *The effect of a mail-mediated intervention on exercise behavior*. US: ProQuest Information & Learning.

Lombard, C., Jolley, D., Deeks, A., & Teede, H. (2008). A low intensity lifestyle intervention prevents weight gain in adult women: a randomized controlled community based intervention. *International Journal of Obesity, 32*, S34.

Loughren, E. A. (2003). *The impact of a fitness/wellness course on psychobehavioral factors*. Eugene, OR; United States: Kinesiology Publications, University of Oregon.

Martin, M. R., & Sharpe, T. L. (2006). Analysis of three exercise adherence interventions on self-efficacy and goal orientation. (abstract). *Research Quarterly for Exercise & Sport, 77*(1), A-81.

Martin, M. Y. (1999). *Increasing physical activity in women who are relatively sedentary*. US: ProQuest Information & Learning.

Mcdonough, M. H. (2007). *The role of relatedness in physical activity motivation, behaviour, and affective experiences: A self-determination theory perspective*. US: ProQuest Information & Learning.

Michalowski, J. R. (1999). *The effects of a stage-matched intervention on physical activity and coronary heart disease risk factors in women*. Eugene, Or.; United States: Microform Publications, University of Oregon.

Miller, K. H., Bryla, K. Y., & Hoke, D. J. (2004). Formative evaluation of a stage-based behavior-change intervention: The university of Kentucky's behavioral health improvement program. (abstract). *Research Quarterly for Exercise & Sport, 75*(1), A-28-a-29.

Monahan, B. (1999). *Effectiveness of a minimal physician delivered stage-based intervention regarding readiness to change specific to physical activity*. Eugene, Or.; United States: Microform Publications, University of Oregon.

Nicolson, A. C. (2001). *A behavioral intervention for shaping the resumption of exercise after drop-out: Implications for practice and theory*. US: ProQuest Information & Learning.

Pattee, D. C. (2000). *The effects of a stage-tailored newsletter on stages-of-change and physical activity levels*. Eugene, OR; United States: Microform Publications, University of Oregon.

Pierce, P. A. (2001). *Effects of a follow-up intervention on the maintenance of physical activity following a fitness course in college-aged women*. US: ProQuest Information & Learning.

Shestopal, A. L. (1999). *Psychological effects of physical exercise and yoga*. US: ProQuest Information & Learning.

Spaziani, M. D. (2003). *Effectiveness of classroom vs. web-based lifetime fitness for health lab instruction on college students' behavioral and psychological physical activity orientation*. Eugene, OR; United States: Kinesiology Publications, University of Oregon.

Steele-Urda, J. L. (2001). *Effects of a stage matched intervention on participation in physical activity among college females*. Eugene, OR; United States: Microform Publications, University of Oregon.

Wadsworth, D. (2006). *Evaluation of a social cognitive theory based e-mail intervention to increase physical activity of college females*. US: ProQuest Information & Learning.

Williams, D. M. (2006). *Multi-phase mediator analysis of a social-cognitive church-based physical activity intervention*. US: ProQuest Information & Learning.

Wilson, D. K. (2008). Commentary for health psychology special issue: Theoretical advances in diet and physical activity interventions. *Health Psychology: Official Journal of the Division of Health Psychology, American Psychological Association, 27*(1), S1-2.

Clinical or elderly Population

Allison, M. J., & Keller, C. (2004). Self-efficacy intervention effect on physical activity in older adults. *Western Journal of Nursing Research, 26*(1), 31.

Bull, S., Eakin, E., Reeves, M., & Riley, K. (2006). Multi-level support for physical activity and healthy eating. *JAN Journal of Advanced Nursing, 54*(5), 585-593.

Burke, V., Giangiulio, N., Gillam, H.F., Beilin, L.J., Houghton, S. (2004). Changes in cognitive measures in a randomized controlled trial of a health promotion program for couples targeting diet and physical activity. *American Journal of Health Promotion, 18*(4), 300-311.

DeJong, S. R., & Veltman, R. H. (2004). The effectiveness of a CNS-led community-based COPD screening and intervention program. *Clinical Nurse Specialist CNS, 18*(2), 72-79.

Dergance, j. M., Mouton, C. P., Lichtenstein, M. J., & Hazuda, H. P. (2005). Potential mediators of ethnic differences in physical activity in older Mexican Americans and European Americans: Results from the San Antonio longitudinal study of aging. *Journal of the American Geriatrics Society, 53*(7), 1240-1247.

Greaney, M. L., Riebe, D., Ewing Garber, C., Rossi, J. S., Lees, F. D., Burbank, P. A., et al. (2008). Long-term effects of a stage-based intervention for changing exercise intentions and behavior in older adults. *The Gerontologist, 48*(3), 358-367.

Greene, B. L., Haldeman, G. F., Kaminski, A., Neal, K., Lim, S. S., & Conn, D. L. (2006). Factors affecting physical activity behavior in urban adults with arthritis who are predominantly African-American and female. *Physical Therapy, 86*(4), 510-519.

Guillot, J., Kilpatrick, M., Hebert, E., & Hollander, D. (2004). Applying the transtheoretical model to exercise adherence in clinical settings. *American Journal of Health Studies, 19*(1), 1-10.

Kuller, L. H., Kriska, A. M., Kinzel, L. S., Simkin-Silverman, L. R., Sutton-Tyrrell, K., Johnson, B. D., et al. (2007). The clinical trial of women on the move through activity and nutrition (WOMAN) study. *Contemporary Clinical Trials, 28*(4), 370-381.

Lippke, S., Ziegelmann, J. P., & Schwarzer, R. (2004). Initiation and maintenance of physical exercise: Stage-specific effects of a planning intervention. *Research in Sports Medicine, 12*(3), 221-240.

Martin, M. Y., Person, S. D., Kratt, P., Prayor-Patterson, H., Kim, Y., Salas, M., et al. (2008). Relationship of health behavior theories with self-efficacy among insufficiently active hypertensive African-American women. *Patient Education and Counseling, 72*(1), 137-145.

Martin, M. Y., Prayor-Patterson, H., Kratt, P., Kim, Y., & Person, S. (2007). Characteristics of insufficiently active hypertensive black women who volunteer to be in a physical activity promotion intervention: An application of social cognitive theory and the transtheoretical model. *Ethnicity & Disease, 17*(4), 604-610.

Moore, S. M., & Charvat, J. M. (2002). Using the CHANGE intervention to enhance long-term exercise. *The Nursing Clinics of North America, 37*(2), 273.

Rejeski, W. J., Brawley, L. R., Ambrosius, W. T., Brubaker, P. H., Focht, B. C., Foy, C. G., et al. (2003). Older adults with chronic disease: Benefits of group-mediated counseling in the promotion of physically active lifestyles. *Health Psychology: Official Journal of the Division of Health Psychology, American Psychological Association, 22*(4), 414-423.

Resnick, B., Luisi, D., & Vogel, A. (2008). Testing the senior exercise self-efficacy project (SESEP) for use with urban dwelling minority older adults. *Public Health Nursing (Boston, Mass.), 25*(3), 221-234.

Richeson, N. E., Croteau, K. A., Jones, D. B., & Farmer, B. C. (2006). Effects of a pedometer-based intervention on the physical performance and mobility-related self-efficacy of community-dwelling older adults: An interdisciplinary preventive health care intervention. *Therapeutic Recreation Journal, 40*(1), 18-32.

Sher, T. G., Bellg, A. J., Braun, L., Domas, A., Rosenson, R., & Canar, W. J. (2002). Partners for life: A theoretical approach to developing an intervention for cardiac risk reduction. *Health Education Research, 17*(5), 597-605.

Sol, B. G. M., van der Graaf, Y., van der Bijl,Jaap J., Goessens, B. M. B., & Visseren, F. L. J. (2008). The role of self-efficacy in vascular risk factor management: A randomized controlled trial. *Patient Education and Counseling, 71*(2), 191-197.

Stephenson, L. E., Culos-Reed, S. N., Doyle-Baker, P. K., Devonish, J. A., & Dickinson, J. A. (2007). Walking for wellness: Results from a mall walking program for the elderly. *Journal of Sport & Exercise Psychology, 29*, S204-S204.

Toobert, D. J., Strycker, L. A., Glasgow, R. E., Barrera Jr, M., & Angell, K. (2005). Effects of the mediterranean lifestyle program on multiple risk behaviors and psychosocial outcomes among women at risk for heart disease. *Annals of Behavioral Medicine: A Publication of the Society of Behavioral Medicine, 29*(2), 128-137.

Van Keulen, H.M., Mesters, I., et al. (2008). Vitalum study design: RCT evaluating the efficacy of tailored print communication and telephone motivational interviewing on multiple health behaviors. *BMC Public Health, 8*.

Van Sluijs,E M F., Van Poppel,M N M., Twisk, J. W. R., Brug, J., & Van Mechelen, W. (2005). The positive effect on determinants of physical activity of a tailored, general practice-based physical activity intervention. *Health Education Research, 20*(3), 345-356.

van Sluijs,Esther M F., van Poppel,Mireille N M., Twisk, J. W. R., & van Mechelen, W. (2006). Physical activity measurements affected participants' behavior in a randomized controlled trial. *Journal of Clinical Epidemiology, 59*(4), 404-411.

van Sluijs, E. M. F., van Poppel, M. N. M., Twisk, J. W. R., Chin A Paw,Marijke J., Calfas, K. J., & van Mechelen, W. (2005). Effect of a tailored physical activity intervention delivered in general practice settings: Results of a randomized controlled trial. *American Journal of Public Health, 95*(10), 1825-1831.

Wiltink, J., Dippel, A., Szczepanski, M., Thiede, R., Alt, C., & Beutel, M. E. (2007). Long-term weight loss maintenance after inpatient psychotherapy of severely obese patients based on a randomized study: Predictors and maintaining factors of health behavior. *Journal of Psychosomatic Research, 62*(6), 691-698.

Woo, J., Sea, M. M. M., Tong, P., Ko, G. T. C., Lee, Z., Chan, J., et al. (2007). Effectiveness of a lifestyle modification programme in weight maintenance in obese subjects after cessation of treatment with orlistat. *Journal of Evaluation in Clinical Practice, 13*(6), 853-859.

No Results

Bredahl, T. V. G., Puggaard, L., & Roessler, K. K. (2008). Exercise on prescription. effect of attendance on participants' psychological factors in a Danish version of exercise on prescription: A study protocol. *BMC Health Services Research, 8*, 139-139.

Cameron, R., Bauman, A., & Rose, A. (2006). Innovations in population intervention research capacity: The contributions of Canada on the move. *Canadian Journal of Public Health.Revue Canadienne De Santé Publique, 97 Suppl 1*, S5-9.

Clark, P. G., Nigg, C. R., Greene, G., Riebe, D., & Saunders, S. D. (2002). The study of exercise and nutrition in older Rhode islanders (SENIOR): Translating theory into research. *Health Education Research, 17*(5), 552-561.

Coday, M., Klesges, L. M., Garrison, R. J., Johnson, K. C., O'Toole, M., & Morris, G. S. (2002). Health opportunities with physical exercise (HOPE): Social contextual interventions to reduce sedentary behavior in urban settings. *Health Education Research, 17*(5), 637-647.

Fortier, (2007). The physical activity counseling (PAC) randomized controlled trial: rationale, methods, and interventions. *Applied Physiology, Nutrition, and Metabolism, 32*(6).

Hogg, W., O’Sullivan, T. L., Blanchard, C., Reid, R. D., Sigal, R. J., Boulay, P., et al. (2007). The physical activity counselling (PAC) randomized controlled trial: Rationale, methods, and interventions. *Applied Physiology, Nutrition & Metabolism, 32*(6), 1170-1185.

Kim, Y. (2008). A stage-matched intervention for exercise behavior change based on the transtheoretical model. *Psychological Reports, 102*(3), 939-950.

King, A. C., Sallis, J. F., Dunn, A. L., Simons-Morton, D. G., Albright, C. A., Cohen, S., et al. (1998). Overview of the activity counseling trial (ACT) intervention for promoting physical activity in primary health care settings. *Medicine & Science in Sports & Exercise, 30*(7), 1086-1096.

King, A.C., Friedman, R., Marcus, B., Castro, C., et al. (2002). Harnessing motivational forces in the promotion of physical activity: the community health advice by telephone project. *Health Education Research, 17*, 627-636,

Lin, J.J., Mamykina, L., Lindtner, S., Delajoux, G., Strub, H.B. (2006). Fish ‘n’ steps: encouraging physical activity with an interactive computer game. *Ubicomp 2006: Ubiquitous Computing, Proceedings, 4206*, 261-278.

Marcus, B.H., Lewis, B.A., et al. (2007). Step into Motion: A randomized trial examining the relative efficacy of internet vs. print-based physical activity intervention. *Contemporary Clinical Trials, 28*, 737-747.

Moe, E. L., Elliot, D. L., Goldberg, L., Kuehl, K. S., Stevens, V. J., Breger, R. K. R., et al. (2002). Promoting healthy lifestyles: Alternative models' effects (PHLAME). *Health Education Research, 17*(5), 586-596.

Peterson, T.R. & Aldana, S.G. (1999). Improving exercise behavior: an application of the stages of change model in a worksite setting. *American Journal of health Promotion, 13*(4), 229-232.

Silva, M.N., Markland, D., et. Al. (2008). A randomized controlled trial to evaluate self-determination theory for exercise adherence and weight control: rational and intervention description. *BMC* *Public Health, 8.*

Slootmaker, S. M., Chin A Paw,Marijke J M., Schuit, A. J., Seidell, J. C., & van Mechelen, W. (2005). Promoting physical activity using an activity monitor and a tailored web-based advice: Design of a randomized controlled trial [ISRCTN93896459]. *BMC Public Health, 5*, 134-134.

Toobert, D. J., Strycker, L. A., Glasgow, R. E., Barrera, M., & Bagdade, J. D. (2002). Enhancing support for health behavior change among women at risk for heart disease: The mediterranean lifestyle trial. *Health Education Research, 17*(5), 574-585.

van Wier, M. F., Ariëns, G. A. M., Dekkers, J. C., Hendriksen, I. J. M., Pronk, N. P., Smid, T., et al. (2006). ALIFE@Work: A randomised controlled trial of a distance counselling lifestyle programme for weight control among an overweight working population [ISRCTN04265725]. *BMC Public Health, 6*, 140-140.

Williams, K., Prevost, A. T., Griffin, S., Hardeman, W., Hollingworth, W., Spiegelhalter, D., et al. (2004). The ProActive trial protocol - a randomised controlled trial of the efficacy of a family-based, domiciliary intervention programme to increase physical activity among individuals at high risk of diabetes [ISRCTN61323766]. *BMC Public Health, 4*, 48-48.

Other reasons

Calfas, K. J., Sallis, J. F., Nichols, J. F., Sarkin, J. A., Johnson, M. F., Caparosa, S., et al. (2000). Project GRAD: Two-year outcomes of a randomized controlled physical activity intervention among young adults. graduate ready for activity daily. *American Journal of Preventive Medicine, 18*(1), 28-37.

Chatzisarantis, N. L. D., & Hagger, M. S. (2005). Effects of a brief intervention based on the theory of planned behavior on leisure-time physical activity participation. *Journal of Sport & Exercise Psychology, 27*(4), 470-487.

Eckstrom, E., Hickam, D. H., Lessler, D. S., & Buchner, D. M. (1999). Changing physician practice of physical activity counseling. *Journal of General Internal Medicine: Official Journal of the Society for Research and Education in Primary Care Internal Medicine, 14*(6), 376-378.

Elavsky, S., & McAuley, E. (2007). Exercise and self-esteem in menopausal women: A randomized controlled trial involving walking and yoga. *American Journal of Health Promotion: AJHP, 22*(2), 83-92.

Hallam, J., & Petosa, R. (1998). A worksite intervention to enhance social cognitive theory constructs to promote exercise adherence. *American Journal of Health Promotion: AJHP, 13*(1), 4-7.

Marcus, B. H., Bock, B. C., Pinto, B. M., Forsyth, L. H., Roberts, M. B., & Traficante, R. M. (1998). Efficacy of an individualized, motivationally-tailored physical activity intervention. *Annals of Behavioral Medicine: A Publication of the Society of Behavioral Medicine, 20*(3), 174-180.

McAuley, E., Blissmer, B., Katula, J., Duncan, T. E., & Mihalko, S. L. (2000). Physical activity, self-esteem, and self-efficacy relationships in older adults: A randomized controlled trial. *Annals of Behavioral Medicine: A Publication of the Society of Behavioral Medicine, 22*(2), 131-139.

Miller, Y. D., Trost, S. G., & Brown, W. J. (2002). Mediators of physical activity behavior change among women with young children. *American Journal of Preventive Medicine, 23*(2), 98-103.

Newton, R. L., & Perri, M. G. (2004). A randomized pilot trial of exercise promotion in sedentary African-American adults. *Ethnicity & Disease, 14*(4), 548-557.

Nichols, J. F., Wellman, E., Caparosa, S., Sallis, J. F., Calfas, K. J., & Rowe, R. (2000). Impact of a worksite behavioral skills intervention. *American Journal of Health Promotion: AJHP, 14*(4), 218.

Pinto, B. M., Lynn, H., Marcus, B. H., DePue, J., & Goldstein, M. G. (2001). Physician-based activity counseling: Intervention effects on mediators of motivational readiness for physical activity. *Annals of Behavioral Medicine, 23*(1), 2-10.

Rejeski, W. J., Shelton, B., Miller, M., Dunn, A. L., King, A. C., & Sallis, J. F. (2001). Mediators of increased physical activity and change in subjective well-being: Results from the activity counseling trial (ACT). *Journal of Health Psychology, 6*(2), 159-168.

Rockwell, T. P. (2002). Stages of change exercise adherence intervention in an un-supervised worksite setting. (abstract). *In Koskolou, M. (ed.), European college of sport science, proceedings of the 7th annual congress of the European college of sport science, Athens, Greece, 24-28 July 2002, Athens, Pashalidis medical publisher, c2002, p.73.* (). Greece:

Sallis, J. F., Calfas, K. J., Alcaraz, J. E., Gehrman, C., & Johnson, M. F. (1999). Potential mediators of change in a physical activity promotion course for university students: Project GRAD. *Annals of Behavioral Medicine, 21*(2), 149-158.

Sallis, J. F., Patrick, K., Calfas, K. J., Zabinski, M. F., Prochaska, J. J., Thompson, S., et al. (1999). A multi-media behavior change program for nutrition and physical activity in primary care: PACE+ for adults. *Homeostasis in Health and Disease, 39*(5), 196-202.

Smith, J. E., Wolfe, B. L., & Laframboise, D. E. (2001). Body image treatment for a community sample of obligatory and nonobligatory exercisers. *The International Journal of Eating Disorders, 30*(4), 375-388.

Steptoe, A., Rink, E., & Kerry, S. (2000). Psychosocial predictors of changes in physical activity in overweight sedentary adults following counseling in primary care. *Preventive Medicine: An International Journal Devoted to Practice and Theory, 31*(2), 183-194.

Wilson, D. K., Friend, R., Teasley, N., Green, S., Reaves, I. L., & Sica, D. A. (2002). Motivational versus social cognitive interventions for promoting fruit and vegetable intake and physical activity in african american adolescents. *Annals of Behavioral Medicine: A Publication of the Society of Behavioral Medicine, 24*(4), 310-319.

Wilson, D. K., Kitzman-Ulrich, H., Williams, J. E., Saunders, R., Griffin, S., Pate, R., et al. (2008). An overview of "the active by choice today" (ACT) trial for increasing physical activity. *Contemporary Clinical Trials, 29*(1), 21-31.
