# Supplementary material for: Mediators of physical activity behaviour change among adult non-clinical populations: a review update
Source: Int J Behav Nutr Phys Act. 2010 May 11;7:37. doi: 10.1186/1479-5868-7-37 (PMC2876989; doi:10.1186/1479-5868-7-37)
Supplement: Additional file 2 — Search syntax. This file contains the search terms used in this review. [file 1479-5868-7-37-S2.DOC]

Additional File 2: Search syntax

MEDLINE search – EBSCOhost, 1998 to September Week 3, 2008

1. Physical activity and psychological theory and intervention
2. Physical fitness and psychological theory
3. Physical fitness and behavioral research
4. Physical fitness and behavioural research
5. Physical activity and behavioural research
6. Physical activity and behavioral research
7. Exercise and behavioral research
8. Exercise and behavioural research
9. Exercise and theoretical effectiveness
10. Physical activity and theoretical effectiveness
11. Physical activity and health behavior and intervention
12. Physical activity and health behavior and intervention – narrowed by age: All Adult 19+ years
13. Physical activity and health behavior and intervention and theory
14. Physical activity and mediator
15. Physical activity and mediator and intervention
16. Exercise and mediator and intervention
17. Exercise and self-efficacy and intervention
18. Exercise and self-efficacy and intervention – narrowed by age: All Adult 19+ years
19. Physical activity and self-efficacy and intervention
20. Physical activity and self-efficacy and intervention – narrowed by age: All Adult 19+ years
21. Physical activity and self-efficacy and intervention and general population
22. Physical activity and self-efficacy and intervention and non-clinical population
23. Physical activity and process of change and intervention
24. Exercise and process of change and intervention
25. Physical activity and Cognitive behavior theory
26. Physical activity and group therapy
27. Physical activity and cognitive process of change
28. Physical activity and cognitive
29. Physical activity and cognitive and intervention
30. Physical activity and cognitive and intervention – narrowed by age: All Adult 19+ years
