# Supplementary material for: Mediators of physical activity behaviour change among adult non-clinical populations: a review update
Source: Int J Behav Nutr Phys Act. 2010 May 11;7:37. doi: 10.1186/1479-5868-7-37 (PMC2876989; doi:10.1186/1479-5868-7-37)
Supplement: Additional file 3 — Review Table of proposed mediators and physical activity behavior. This file contains data extracted from each included article in table form for quick reference. [file 1479-5868-7-37-S3.DOC]

Additional File 3

Title: Review Table of proposed mediators and physical activity behaviour

| Authors | Participants | Intervention | Constructs measured | PA measure | Behaviour Change | Action Theory Test | Conceptual Theory Test | Mediated Effect | Quality of studies /11 |
| --- | --- | --- | --- | --- | --- | --- | --- | --- | --- |
| Ash et al. (2006) | 176 adults with BMI >27 kg/m^2^ | RCT, 12 months, real practice setting  3 arms: 1^st^-group based CBT lifestyle diet and exercise sessions; 2^nd^-individualised dietetic treatment; control-nutrition resource booklet | self-efficacy | IPAQ | Non significant (ns) and trivial effect size (ES) | Generalized Self-efficacy  (between intervention groups and control) at 3 months and 12 months  ES=small | Not stated | Not stated | 6 |
| Bennet et al. (2008) | 72 inactive adults | RCT, 6 months, rural home setting, 2 arms: int. group-motivational interviewing phone calls; control-phone calls with no MI content | self-efficacy | CHAMPS physical activity questionnaire for older adults | No (ns) and trivial ES | Self-efficacy  ES=medium | Not stated | Not stated | 5 |
| Bock et al. (2001) | 150 sedentary adults | RCT, 6 months, 2 arms: int. group-individualized motivationally tailored print materials; control-standard exercise promotion print materials  Assessment at 12 month follow-up | **TTM**  perceived barriers and benefits, self-efficacy, processes of change | 7Day PAR | No (ns) and trivial ES | No (ns) | Self-efficacy, barriers, and behavioural processes sig | Not stated | 6 |
| Cardinal & Spaziani (2007) | 109 University students | Quasi-experimental design, 10 weeks, 3 arms: 1^st^ lifestyle class, 2^nd^ online lifestyle class, 3^rd^ control classes | **TTM**  perceived barriers and benefits, self-efficacy, processes of change | GLTEQ | No (ns) | No (ns) | Not stated | Not stated | 4 |
| Cerin et al. (2006) | 52 inactive adults | RCT, 16 weeks, 2 arms: 1st group-print only; 2^nd^ group-print plus telephone calls to encourage/assess progress. Assessment at 16 weeks and 4 week follow-up | Social support | CHAMPS | Yes – sig change at 16 weeks (small ES). NS difference at follow-up and trivial ES | Social support at 16 weeks  (ES=small) but not at follow-up | Yes – change at 16 weeks (small ES). No (ns) difference at follow-up and trivial ES | Social support was a mediator at 16 weeks. (small ES) | 8 |
| Cramp & Brawley (2006) | 57 post natal women | RCT, 4 weeks, 2 arms: 1st group-standard exercise; 2^nd^ group-mediated by group cognitive behavioural counselling. Assessment at 4 weeks and 4 week follow-up | **SCT**  Outcome Expectations,  Barrier Self-efficacy | PAR | Yes – sig change at 4 and 8 weeks in favour of the group-mediated intervention (large ES) | Yes – change in outcome expectations and barrier self-efficacy were sig higher in the group mediated intervention (large ES) | Not stated | Not stated | 5 |
| Dallow & Anderson (2003) | 58 sedentary obese women | RCT, 48 weeks, 2 arms: int. group-theory based lifestyle group sessions; control-free access to fitness facility. Assessments at 24 and 48 weeks. | **TTM**  Processes of change, self-efficacy | 7 Day PAR | Yes - sig difference for lifestyle at 24 weeks  (ES=medium) and 48 weeks (ES = large). | -self-reevaluation and environmental reevalaution were sig higher in lifestyle group at 24 weeks (medium ES). NS differences at 48 weeks. | Not stated | Not stated | 5 |
| Dinger et al. (2007) | 56  insufficiently active women | 2 group experimental, 6 weeks, both groups received pedometers, step logs, print, weekly emails; one group also received emails based on TTM constructs | **TTM**  Processes of change, decisional balance, self-efficacy | IPAQ | No (ns) | No (ns) | Not stated | Not stated | 5 |
| Elbel et al. (2003) | 120 skilled labour employees | Quasi experimental at 3 work sites, 4 weeks. 1^st^ site professional education sessions based on TTM/SCT, 2^nd^ site peer-led education sessions based on TTM, 3^rd^ site control. Assessments post-test and 4 weeks follow-up. | Self-efficacy | PAR | No (ns) | No (ns) | Not stated | Not stated | 4 |
| Fahrenwald et al. (2004, 2005) | 44 sedentary mothers with children | RCT, 10 weeks, WIC setting, 2 arms: int. group-counselling and biweekly phone calls focusing on PA; control-counselling and phone calls focusing on self-breast examination | **TTM**  decisional balance, self-efficacy, self-liberation, counterconditioning, environmental reevalaution, social support (SS) | 7 Day PAR | Yes  ES=large | All constructs sig | All constructs sig | Test did not support mediation | 7 |
| 1. Fortier et al. (2007)  2. Blanchard et al. (2007) | 120 inactive adults | RCT, 13 weeks, primary care practice setting, 2 arms: int. group-counselling from HCP plus intensive autonomy support PA counselling; control-counselling from HCP | **1.SDT**  autonomy support, autonomy, perceived competence  2. barrier self-efficacy, task self-efficacy | GLTEQ | Yes  ES=large | 1. autonomy support  -autonomous motivation ES=small  Perceived competence was NS  2. task self-efficacy and barrier self-efficacy (ES = medium) | 1. Autonomy support sig but not autonomous motivation or competence2. task and barrier self efficacy were sig | 1.Not stated  2. task self-efficacy was a sig partial mediator (small ES); barrier efficacy was a sig mediator but trivial ES | 8 |
| Gallagher et al. (2006) | 165 overweight women | RCT, 6 months, home setting, 4 arms: all groups received weekly sessions; assigned to 1000 kcal/wk at MPA; 1000 kcal/wk at VPA; 2000 kcal/wk at MPA; 2000 kcal/wk at VPA | **TTM**  self-efficacy,  decisional balance, expected outcomes and barriers, processes of change | 7 Day PAR | No (ns) | No (ns) | Sig for self-efficacy, decisional balance, processes of change | Not stated | 6 |
| Hallam & Petosa (2004) | 82 adult employees | non-equivalent pre-post test repeated measures, 12 months, workplace setting, 2 arms: int. group-counselling sessions plus access to on-site fitness facility; control-new members of fitness center | **SCT**  outcome expectancy, self-regulation, self-efficacy | 7 Day recall | No (ns) difference at 6 weeks, 6 months, but sig at 12 months | -self-regulation (ES=large)  -outcome expectancy (ES=small)  No (ns) difference in self-efficacy | Not stated | Possible mediation found for self-regulation at 12 months, not found for outcome expectancy | 5 |
| Hurling et al. (2007) | 77 adults | stratified control trial, 9 weeks, 2 arms: int. group-internet based behaviour change system, with email or mobile phone reminders; control-verbal advice on PA only | perceived control, intention/motivational change – all created for this study | IPAQ and blue  tooth accelerometer | Yes - sig differences between groups on both measures | Sig differences perceived control  intention  internal control/external control (instruments created for study) but not motivational change (instrument created for study) | Not stated | Not stated | 6 |
| Jacobs et al. (2004) | 511 low income women | Non-random assignment , 1 year, 2 arms: 1^st^ group intensive counselling and computer intervention based on TTM and SCT, 2^nd^ group minimal health advice standard. | Self-efficacy, perceived barriers, social support | Questionnaire created for the study | No (ns) | No (ns) | No (ns) | Not stated | 4 |
| Jones et al. (2004) | 450 psychology students | RCT, 2 weeks, university setting, 6 arms: participants received positively or negatively framed pamphlets and were told materials were from 1) credible source, 2) non credible source, 3) no source reported | **TPB**  behavioural beliefs, exercise attitudes, subjective norm, perceived behavioural control, exercise intention | GLTEQ | No (ns) | No (ns) differences for any TPB variable | Not stated | Not stated | 6 |
| Kinmonth et al. (2008) | 365 sedentary adults | RCT, 1 year, GP setting, 3 arms: 1^st^ theory-based behaviour change program at home, 2^nd^ theory based program by phone, 3^rd^ was a control group given a brief advice leaflet. | Intention | Heart Rate, corroborated by Vo2 max testing; EPIC Norfolk physical activity questionnaire | No (ns) and trivial ES | Change in intention in favour of the intervention groups at six months (medium ES) but no (ns) difference at one year. | Not stated | Not conducted | 7 |
| Kloek et al. (2006) | 1926 adults | Quasi-experimental, 2 year, community setting, 2 arms: int. communities-given action plans related to determinants of health, courses, and special events; control-comparison communities | Unspecified theory  -attitude, self-efficacy, | SQUASH | No (ns) | No (ns) | Not stated | Not stated | 3 |
| Levy & Cardinal (2004) | 126 sedentary adults with intention of starting an exercise program | RCT, 2 months, community setting, 3 arms: 1^st^ int. group-mail delivered packet promoting SDT constructs; 2^nd^ int. group-packet plus booster postcard; control-PA facts booklet | **SDT**  perceptions of autonomy, perceptions of competence, perceptions of relatedness, behavioural regulation | Leisure Time Exercise Questionnaire | No (ns) | No (ns) | Not stated | Not conducted because of the null effects | 6 |
| Lewis et al. (2006) | 150 sedentary adults | RCT, 6 months, 1^st^ group received motivationally tailored intervention, 2^nd^ group received generalized exercise intervention. Assessments at 1,3, and 6 months. | **TTM**  Processes of change, self-efficacy, decisional balance | PAR | Yes – sig difference in favour of motivationally tailored group (small ES) | Significant effect on behavioural processes (small ES) but not on Cognitive processes, self-efficacy, decisional balance | Sig effect on self-efficacy and behavioural processes but ns for cognitive processes and decisional balance | Behavioural processes of change did not act as a sig mediator | 9 |
| Little et al. (2004) | 151 sedentary adults | RCT, 1 month, practice setting, 8 arms: 1^st^ int. group-Health Education Authority booklet; 2^nd^ int. group-counselling sessions; 3^rd^ int. group-exercise prescription by GP. Participants could received no int., a single int., or a combination of all 3 | unspecified theory  -stage of change,  intention, perceived behavioural control, attitude, attitude of important others, attitude of important others to practical help | GLTEQ | Yes – sig only in most intensive group-counselling plus exercise prescription | Intention different among groups for those with low intention at baseline.[1-6] | Intention was related to behaviour | Not stated | 3 |
| Milne et al. (2002) | 248 undergraduate students | Post-test design, 2 weeks, university setting, 3 arms: 1^st^ int. group-motivational leaflet; 2^nd^ int. group-motivational leaflet plus volitional int.; control-neither, read 3 paragraphs of a novel | **PMT**  perceived severity, perceived vulnerability, response efficacy, self-efficacy, response costs | PA questionnaire, number of 20 minute sessions in past week | Yes - sig group 2 increased PA compared to group 1 and control  ES=medium | Yes – sig differences between int. groups and control for perceived vulnerability, perceived severity of premature death, perceived severity of pain, fear, response efficacy, self-efficacy, response costs, intention | Not stated | Not stated | 5 |
| Napolitano et al. (2008) | 239 inactive adults | RCT, 6 months, 3 arms: 1^st^ print-based motivationally tailored, 2^nd^ telephone-based motivationally tailored, 3^rd^ contact control. Assessments at 6 mos and 12 mos follow-up. | **TTM**  Processes of change, self-efficacy, decisional balance | PAR | Yes –both intervention groups positively changed physical activity (medium ES) | All constructs (medium to large ES) | Behavioural processes were sig all other variables were ns | Processes (behavioural) were supported as as a mediator. Cognitive processes acted as a suppressor. | 8 |
| Parrott et al. (2008) | 170 sedentary college students | Randomized groups pre-post test, 2 weeks, university setting, 3 arms: 1^st^ int. group-positively framed emails; 2^nd^ int. group-negatively framed emails; control-no emails | **TPB**  attitude, intention,  subjective norm, perceived behavioural control | GLTEQ | Yes-Positively framed sig different than control group. | Yes - intention (positively and negatively framed higher than control group)  -affective attitude (positively and negatively framed higher than control group when baseline was not low)  -perceived behavioural control(positively and negatively framed higher than control group) . No (ns) for Instrumental attitude and subjective norm | Not tested | Not stated | 6 |
| Plotnikoff et al. (2005) | 2121 employees | pre-post test design, 12 weeks, workplace setting, 2 arms: int. group-weekly email message; control-no email | **TTM, PMT**  self-efficacy, pros and cons, severity, intention | GLTEQ | Yes- sig difference between groups but trivial effect size | No (ns) | Not stated | Not stated | 6 |
| Reger et al. (2002) | 31,420 sedentary adults aged 50-65 | Quasi-experimental 8wk community study, target community received paid media and public relations communications for walking, control community no communication | **TPB**  Attitude, subjective norm, PBC, intention | Observation and self-report measure | No (ns) and trivial effect size | No (ns) for attitude or subjective norm. Sig differences in intention and PBC but trivial effect sizes | Not stated | Not stated | 5 |
| Rovniak et al. (2005) | 50 sedentary women | RCT, 12 weeks, 2 arms: 1^st^ int. group-high theoretical fidelity email messages; 2^nd^ int. group-low theoretical fidelity email messages. Included a 1 yr follow-up. | **SCT**  self-efficacy, outcome expectations, enjoyment, goal setting and planning, social support | self-report logs and questionnaire | No (ns) at 12 weeks and 1 yr and trivial effect size | Yes- sig for goal setting (ES=med), positive outcome expectation (ES=med) | Not stated | Not stated | 6 |
