# Supplementary material for: Mediators of physical activity behaviour change among adult non-clinical populations: a review update
Source: Int J Behav Nutr Phys Act. 2010 May 11;7:37. doi: 10.1186/1479-5868-7-37 (PMC2876989; doi:10.1186/1479-5868-7-37)
Supplement: Additional file 4 — Data extraction. This file contains the data extracted from each included article. [file 1479-5868-7-37-S4.DOC]

Additional File 4

Title: Data extraction

**Study – Ash et al. (2006)**

**Sample –** 176 adults with BMI >27 kg/m^2^

**Design –** RCT, 3 arm, 7 measures

**Setting –** real practice setting

**PA target –** 20-30 min sessions most days/week

**DV –** International physical activity questionnaire: amount of time spent on activity in past 7 days (test-retest reliability Spearman’s correlation coefficient was 0.80); anthropometric variables

**Intervention theory, length, type –** Self-efficacy, 12 months, 1^st^ intervention: group-based lifestyle intervention, group weekly sessions for 8 weeks, monthly sessions for 6 months (Fat Booters Incorporated); 2^nd^ intervention group: individualised dietetic treatment, 8 weekly individual contacts, monthly sessions for 6 months; control group: given no further advice than nutrition resource booklet

**Outcomes –** PA at 3 months: OR of being sufficiently active in relation to Fat Booters Incorporated group: 0.19 times lower in control, 0.27 times lower in individualised dietetic treatment. No differences at 12 months (ns) and trivial effect size.

**Mediating Variables –** Differences in generalized self-efficacy at 3 months and 12 months. ES=small

**Mediator Analysis –** none stated

**Study – Bennet et al. (2008)**

**Sample –** 72 inactive adults

**Design –** RCT, 2 arm, 3 measure

**Setting –** rural home setting

**PA target –** 30 minutes of moderate intensity most days/week

**DV –** PA: CHAMPS physical activity questionnaire for older adults (scores converted into caloric expenditure per week)

**Intervention theory, length, type –** self-efficacy, 6 months, Intervention group: received pedometers and monthly motivational interviewing (MI) calls. Control group: received phone calls with no MI content.

**Outcomes –** PA: No differences between baseline and 6 months for either group and trivial effect size

**Mediating Variables –** Differences in self-efficacy for intervention group. ES=medium; No differences between groups for stages of change

**Mediator Analysis –** none stated

**Study – Bock et al. (2001)**

**Sample –** 150 sedentary adults

**Design –** RCT, 2 arm, 6 measures

**Setting -** not stated

**PA target –** 30 minutes of moderate-intensity activity at least 5 days per week

**DV –** PA maintenance during 6 months following the intervention period: 7 day PAR.

**Intervention theory, length, type –** TTM, 6 months, 1^st^ intervention group: individualized, motivationally tailored print materials (IT); 2^nd^ intervention group: standard exercise promotion print materials (ST).

**Outcomes –** PA maintenance: IT participants reported more time spent in PA per week at month 12 (187 minutes ) than ST group (133 minutes), but differences were not significant at 12 months (trivial ES)

**Mediating variables –** No differences in self-efficacy, positive mood, use of behavioural processes of change, perceived benefits or barriers to PA, depressive symptoms, and use of cognitive processes at 12 months (trivial ES).

**Mediator Analysis –** none stated

**Study – Cardinal & Spazani (2007)**

**Sample** – 109 university students

**Design** – 10 week quasi-experimental, 3 arm, 4 measures

**Setting** – university

**PA target** – not stated

**DV** – PA: GLTEQ

**Intervention theory, length, type**: TTM, 10 weeks, 1^st^ group: lifestyle class; 2^nd^ group: online lifestyle class; 3^rd^: control classes.

**Outcomes** – PA: no differences across groups.

**Mediating variables** – no difference across groups for perceived barriers and benefits, self-efficacy, POC

**Mediator Analysis** – none stated

**Study – Cerin et al. (2006)**

**Sample –** 52 inactive adults

**Design –** RCT, 2 arm, 2 measures

**Setting –** not stated

**PA target –** 30 minutes or more of moderate intensity most days/week

**DV –** PA: self-report frequency and duration of high, moderate, and low activity over past 4 weeks

**Intervention theory, length, type –** Social support, 16 weeks, intervention groups received counselling, instructional newsletters, pedometers, weekly activity logs. 1^st^ intervention group: print only. 2^nd^ intervention group: print plus weekly telephone calls to encourage and assess participant’s progress.

**Outcomes –** PA: change at 16 weeks (small ES). No difference at follow-up (trivial ES)

**Mediating Variables –** Mediation effect of social support on initial behaviour change at 16 weeks but not on maintenance. ES=small

**Mediator Analysis –** 4 methods of mediation analysis (Baron-Kenny, Freedman-Schatzkin, MacKinnon et al, bootstrap method). Social support was a mediator at 16 weeks, as indicated by Freedman-Schatzkin, MacKinnon et al, bootstrap method. (small ES)

**Study - Cramp & Brawley (2006)**

**Sample –** 57 post natal women

**Design** – RCT, 2 arm, 4 measures

**Setting** – fitness facility and home

**PA** **target** – daily accumulation of mild to moderate PA three or more days per week.

**DV** – PA: 7-day PAR assessed self-reported PA, which has been validated using objective methods.

**Intervention theory, length, type** – SCT, 4 weeks, 1st group-standard exercise; 2^nd^ group-mediated by group cognitive behavioural counselling. Assessment at 4 weeks and at 4 week follow-up.

**Outcomes** – PA: significant change at 4 and 8 weeks in favour of the group-mediated intervention (ES=large)

**Mediating variables** – change in outcome expectations and barrier self-efficacy were significantly higher in the group mediated intervention (ES=large).

**Mediator Analysis** – none stated

**Study – Dallow & Anderson (2003)**

**Sample –** 58 sedentary obese women

**Design –** RCT, 2 arm, 4 measures

**Setting –** usual care group: recreational facility; lifestyle group: not stated

**PA target –** 30 min of moderate-intensity activity 4 or more days/week

**DV –** PA: PAR (estimates energy expenditure in PA during a 7 day period, correlations b/w PAR and Tritrac R-3D regression equation b/w r=.86 and r=.95 across 7 days), Fitness: estimated maximal oxygen uptake for bicycle ergometry

**Intervention theory, length, type –** TTM, 48 weeks, Intervention group: cognitive and behavioural theory based lifestyle group, met weekly for 16 weeks and bi-weekly for remaining 8 weeks; Control: usual care group with free access to fitness facility

**Outcomes –** PA: Significant difference for lifestyle at 24 weeks (ES=medium) and at 48 weeks (ES = large).

**Mediating Variables –** self-reevaluation and environmental reevalaution were significantly higher in lifestyle group at 24 weeks (medium ES). No different at 48 weeks. Change in Behavioural processes, consciousness raising, dramatic relief, social liberation and self-efficacy were not different across groups.

**Mediator Analysis -** none stated

**Study – Dinger et al. (2007)**

**Sample –** 56 insufficiently active women

**Design –** 2 group experimental, 4 measures

**Setting –** not specified

**PA target –** 30 minutes of moderate-intensity PA most days of the week

**DV –** PA: IPAQ to assess minutes spent walking during the last 7 days. Criterion validity (*p*=.30) and test-retest reliability (*p*=.75)

**Intervention theory, length, type –** TTM, 6 weeks, both intervention groups received pedometers, step logs, and received weekly email reminders. One group also received emails using TTM strategies to increase PA.

**Outcomes –** PA: no differences between groups

**Mediating Variables –** No differences between groups with all TTM variables

**Mediator Analysis –** none stated

**Study – Elbel et al. (2003)**

**Sample –** 120 skilled labour employees

**Design** – Quasi experimental at 3 work sites, 3 measures

**Setting** – worksite

**PA** **target** – vigorous activity performed 3 or more times per week for at least 20 minutes, or moderate activity 5 or more times per week for at least 30 minutes.

**DV** – PA: 7 day PAR administered in a group format

**Intervention theory, length, type** – self-efficacy, 4 weeks, 1^st^ site professional education sessions based on TTM/SCT, 2^nd^ site peer-led education sessions based on TTM, 3^rd^ site control. Assessments post-test and 4 weeks follow-up

**Outcomes** – No differences between groups

**Mediating** **variables** – No differences in self-efficacy between groups.

**Mediation** **Analysis** – none stated

**Study – Fahrenwald et al. (2004; 2005)**

**Sample –** 44 sedentary mothers with children

**Design –** 2 group experimental, 6 measures

**Setting –** Women, Infants, and Children program sites

**PA target –** 30 minutes of brisk activity performed 5 days/week or more

**DV –** PA: 7 Day PAR to assess past week’s PA energy expenditure (Cronbach’s alpha=.77)

**Intervention theory, length, type –** TTM, 10 weeks, 1^st^ intervention group: counselling and 4 biweekly telephone contacts with focus on PA, 2^nd^ group: counselling and 4 biweekly telephone contacts with focus on self-breast examination.

**Outcomes –** PA: sig. increases in experimental group (ES=large)

**Mediating Variables –** Changes in self-efficacy, pros, cons, decisional balance, self-liberation, counterconditioning, environmental reevaluation, and social support. (ES for all variables=large)

**Mediator Analysis** – Unspecified test did not support mediation. Sobel test not reported for partial mediation.

**Study – 1) Fortier et al. (2007); 2) Blanchard et al. (2007)**

**Sample -** 120 adults reporting less than 150 minutes of PA/week

**Design –** RCT, 2 arm, 4 measure

**Setting –** primary care practice

**PA target -** >150 minutes of moderate intensity activity per week

**DV –** PA: GLTEQ to assess self-report PA that last for longer than 20 minutes in a typical week. Has a strong relationship with objective measures such as the activity monitor (r=.45) and fitness test (r=.56)

**Intervention theory, length, type –** 1) SDT, 13 weeks, intervention group: counselling from HCP plus intensive autonomy supportive PA counselling biweekly for 3 months. Control group: counselling from HCP only. 2) 2. barrier self-efficacy, task self-efficacy

**Outcomes –** PA: significant differences in experimental group from baseline (15.54) to 13 weeks (26.74). (ES=large)

**Mediating Variables –** 1) sig. differences for autonomy support and autonomous motivation. (ES=small) No differences for perceived competence. 2. task self-efficacy and barrier self-efficacy (ES = medium)

**Mediator Analysis –** 1) none stated. 2) 2. Krull and MacKinnon procedure - task self-efficacy was a significant partial mediator (small ES); barrier efficacy was a significant mediator but ES was trivial

**Study – Gallagher et al. (2006)**

**Sample –** 165 overweight women ages 21-45

**Design –** RCT, 4 arm, 6 measures

**Setting –** home based exercise program

**PA target -** >150 minutes per week

**DV –** PA: interviewer administered 7day PAR to assess frequency and time spent in sedentary, light, moderate, or vigorous PA over previous 7 days.

**Intervention theory, length, type –** TTM, 6 months, weekly group sessions to address behavioural strategies to reduce energy intake and increase energy expenditure. Participants randomly assigned to 1) 1000 kcal/wk at moderate intensity 2) 1000 kcal/wk at vigorous intensity 3) 2000 kcal/wk at moderate intensity 4) 2000 kcal/wk at vigorous intensity

**Outcomes –** PA: no differences between groups

**Mediating Variables –** no differences between groups in all TTM variables.

**Mediator Analysis –** none stated

**Study – Hallam & Petosa (2004)**

**Sample –** 82 participants

**Design –** non-equivalent pretest/posttest repeated measures, 2 arm, 4 measures

**Setting -** workplace

**PA target –** 3 days/week

**DV –** PA: 7 day recall paper-pencil questionnaire to asses number of days and minutes engaged in exercise behaviour over the previous 7 days.

**Intervention theory, length, type –** SCT, 12 months. Intervention group: attended four 1 hr session to increase the use of self-regulation skills, dispelling the myths of exercise, identifying the expected outcomes from exercise participation, and teaching how to engage in a safe, efficient, and effective exercise program. Treatment group had access to the company’s on-site fitness facility. Comparison group: joined regular fitness center programs no longer than 30 days prior to the beginning of the intervention.

**Outcomes –** PA: No difference at 6 weeks, 6 months, but significant at 12 months

**Mediating Variables –** Self-regulation: sig. differences between groups. Intervention group sig. increased self-regulation from baseline (97.20) to 12 month (121.13), comparison group sig. decreased from 109.68 to 100.82 (ES=large). Outcome expectancy: sig. increase in intervention group from baseline (111.50) to 12 month (127.98), sig. decrease in comparison group from baseline (141.29) to 12 month (120.71) (ES=small). Self-efficacy: no sig. difference between groups.

**Mediator Analysis –** Unspecified test found mediation for self-regulation at 12 months, not found for outcome expectancy

**Study – Hurling et al. (2007)**

**Sample –** 77 adults

**Design –** stratified controlled trial, 2 arm, 4 measures

**Setting –** not specified

**PA target –** at least 30 minutes most days/week

**DV –** PA: IPAQ self-report and Bluetooth wrist-worn accelerometer

**Intervention theory, length, type –** not specified, 12 weeks, intervention group: used internet based behaviour change system with unlimited access for 9 weeks. Control: received verbal advice on PA recommendations only.

**Outcomes –** PA: Significant differences between groups on both measures

**Mediating Variables –** Significant differences in perceived control, intention/expectation, internal control, and external control (instruments created for study). No sig. differences for motivational change (instrument created for study).

**Mediator Analysis –** none stated

**Study – Jacobs et al. (2004)**

**Sample –** 511 low income women

**Design** – non-random assignment, 2 arm, 4 measures

**Setting** – health departments

**PA** **target** – 30 minutes of activity on most days

**DV** – PA: questionnaire created for study

**Intervention theory, length, type** – 1 year, 1^st^ group intensive counselling and computer intervention based on TTM and SCT, 2^nd^ group minimal health advice standard.

**Outcomes** – PA: no differences between groups

**Mediating** **variables** – no differences between groups

**Mediator** **Analysis** – none stated

**Study – Jones et al. (2004)**

**Sample –** 450 psychology students

**Design –** RCT, 6 arm, 8 measures

**Setting -** university

**PA target –** 30 minutes of vigorous intensity 3 or more times/week

**DV –** PA: Godin Leisure Time Exercise Questionnaire measuring leisure time exercise sessions ≥30 minutes.

**Intervention theory, length, type –** TPB, 2 weeks, participants received pamphlet promoting healthy lifestyles and active living. Credible source group: pamphlet perceived to be from medical doctor. Non credible source: pamphlet perceived to be from high school student. Control group: no source reported. Groups could either receive positively or negatively framed messages.

**Outcomes –** PA: No significant differences across groups

**Mediating Variables –** TPB variables: no differences.

**Mediator Analysis –** none stated

**Study – Kinmonth et al. (2008)**

**Sample** – 365 sedentary adults

**Design** – RCT, 3 arm, 6 measures

**Setting** – GP setting

**PA** **target** – not stated

**DV** – PA: Heart Rate, corroborated by Vo2 max testing; EPIC Norfolk physical activity questionnaire

**Intervention, length, type** – not specified, 1 year, 1^st^ theory-based behaviour change program at home, 2^nd^ theory based program by phone, 3^rd^ was a control group given a brief advice leaflet.

**Outcomes** - No significant difference for change across groups on any measure (trivial ES).

**Mediating** **Variables** – Change in intention in favour of the intervention groups at six months (medium ES) but no difference at one year

**Mediator** **Analysis** – none conducted

**Study – Kloek et al. (2006)**

**Sample –** 1926 adults

**Design –** quasi-experimental, 2 arm, 7 measures

**Setting -** community

**PA target –** 30 minutes of moderate physical activity at least 5 days per week

**DV –** PA: SQUASH questionnaire to assess frequency and duration of activity in an average week. Spearman correlation coefficient for reproducibility=0.58 and relative validity=0.45.

**Intervention theory, length, type –** no specific theory, 2 year, intervention communities: given action plans related to determinants of health, face-to-face courses and one-off special events. Control group was comparison communities.

**Outcomes –** PA: no sig. differences within or between groups.

**Mediating Variables –** No differences for physical activity attitude or physical activity self-efficacy.

**Mediator Analysis –** none stated

**Study – Levy & Cardinal (2004)**

**Sample –** 126 sedentary adults with intention of starting an exercise program

**Design –** RCT, 3 arm, 6 measures

**Setting -** community

**PA target –** 3 times/week on a regular basis

**DV –** PA: Leisure Time Exercise Questionnaire to assess self-reported frequency and intensity of PA during a typical week for periods of 15 minutes of longer. Test-retest = 0.74.

**Intervention theory, length, type –** SDT, 2 months, 1^st^ intervention group: mail delivered packet of behaviour and cognitive strategies promoting SDT constructs. 2^nd^ intervention group: packet and booster postcard 1 month later. Control: received American Heart Association PA facts booklet.

**Outcomes –** PA: no differences between groups.

**Mediating Variables –** No differences in autonomy, behavioural regulation, competence, or relatedness.

**Mediator Analysis –** none conducted because of the null effects

**Study – Lewis et al. (2006)**

**Sample –** 150 sedentary adults

**Design** – RCT, 2 arm, 5 measures

**Setting** – not stated

**PA** **target** – vigorous activity less than 3 times a week for 20 minutes or moderate activity less than 5 days a week for 30 minutes

**DV** – PA: 7 day PAR

**Intervention theory, length, type** – TTM, 6 months, 1^st^ group received motivationally tailored intervention, 2^nd^ group received generalized exercise intervention. Assessments at 1,3, and 6 months.

**Outcomes** – PA: difference in favour of motivationally tailored group (small ES)

**Mediating** **variables** – Significant effect on behavioural processes (small ES). No differences in cognitive processes, self-efficacy, decisional balance

**Mediator** **Analysis** – Baron and Kenny - Behavioural processes of change did not act as a significant mediator

**Study – Little et al. (2004)**

**Sample –** 151 sedentary patients with risk factors for cardiovascular disease

**Design –** RCT, 8 arm, 11 measures

**Setting –** 4 general practices (GP)

**PA target –** 30 minutes of brisk exercise 5 days/week not requiring a leisure facility (i.e.-walking)

**DV –** PA: Godin questionnaire which multiplies the number of episodes of exercise by relative energy expenditure. Fitness: 6 minute walk test (correlations to VO2 max are between 0.6-0.8).

**Intervention theory, length, type –** unspecified psychological and behavioural theory, 1 month, participants were assigned to one of 8 possible groups: no intervention, a single intervention, or any combination of intervention. 3 intervention factors: Health Education Authority booklet, counselling sessions based on attitudes and perceived behavioural control, or exercise prescription by a GP.

**Outcomes –** PA and fitness: sig changes from baseline only in most intensive group (prescription and counselling)

**Mediating Variables –** No differences found in all psychosocial variables.

**Mediator Analysis –** none stated

**Study – Milne et al. (2002)**

**Sample –** 248 undergraduate students

**Design –** post-test design, 3 groups, 3 measures

**Setting –** university

**PA target –** at least 20 minutes, with a noticeable increase in heart rate, per week

**DV –** PA questionnaire assessing the number of sessions totalling ≥20 minutes of PA in the past week.

**Intervention theory, length, type –** PMT, 2 weeks, 1^st^ intervention group: received only motivational intervention leaflet. 2^nd^ intervention group: received both the motivational intervention leaflet and the volitional intervention, which asked them when and where they would carry out exercise in the following week. Control group: received neither intervention, and were instead asked to read the first 3 paragraphs of a novel.

**Outcomes -** PA: group 2 engaged in sig. more PA than group 1 or control. No sig. differences between group 1 and control. At the end of the 2 week intervention, 38% of control, 35% of group 1, and 91% of group 2 engaged in exercise. (ES=medium)

**Mediating variables –** Sig. differences between experimental groups and control for perceived vulnerability, perceived severity of premature death, perceived severity of pain, fear, response efficacy for lessen chances if CHD and reduce risk of CHD, self-efficacy, response costs, and intention. No differences between intervention groups 1 and 2.

**Mediator Analysis –** none stated

**Study – Napolitano et al. (2008)**

**Sample –** 239 inactive adults

**Design** – RCT, 3 arm, 4 measures

**Setting** – not stated

**PA** **target** – participating for greater than 90 minutes of moderate to vigorous activity per week.

**DV** – PA: 7 day PAR (test-retest reliability 0.86)

**Intervention, length, type** – TTM, 6 months, 1^st^ print-based motivationally tailored, 2^nd^ telephone-based motivationally tailored, 3^rd^ contact control. Assessments at 6 months and 12 months follow-up

**Outcomes** – PA: Both intervention groups positively changed physical activity (medium ES)

**Mediating** **variables** – Differences in POC, self-efficacy, decisional balance (medium to large ES)

**Mediator** **Analysis** – Baron and Kenny - Processes (behavioural) were supported as as a mediator. Self-efficacy and decisional balance were not. Cognitive processes acted as a suppressor

**Study – Parrott et al. (2007)**

**Sample –** 170 sedentary college students

**Design –** randomized groups pre-post test, 3 arm, 4 measure

**Setting –** not stated

**PA target –** 30 minutes moderate or vigorous intensity ≥5 days/week

**DV –** PA: GLTEQ to assess duration and intensity of exercise during free time. 2 week test-retest reliability r=.74

**Intervention theory, length, type –** TPB, 3 weeks, 1^st^ intervention group: positively framed emails every other day for 2 weeks (PFM). 2^nd^ intervention group: negatively framed messages every other day for 2 weeks (NFM). 3^rd^ group: control.

**Outcomes –** PA: Positively framed significantly different than control group.

**Mediating Variables –** Differences in intention (positively and negatively framed higher than control group), affective attitude (positively and negatively framed higher than control group when baseline was not low), perceived behavioural control (positively and negatively framed higher than control group). No differences in instrumental attitude and subjective norm.

**Mediator Analysis –** none stated

**Study – Plotnikoff et al. (2005)**

**Sample –** 2121 employees with access to personal email

**Design –** pre and post test design, 2 arm, 6 measures

**Setting –** 5 large workplaces

**PA target –** Health Canada’s current recommended guidelines

**DV –** PA: GLTEQ measure weekly energy expenditure; Diet: 5 point scale

**Intervention theory, length, type –** TTM, PMT, 12 weeks, intervention group: one PA and one nutrition email message/week. Control group: no weekly message

**Outcomes –** PA: intervention group increased MET total minutes from 664.05 to 683.68. Control sig. decreased from 668.56 to 592.66. Significant difference between groups but trivial effect size

**Mediating Variables –** differences in self-efficacy, behavioural intention, pros, cons, but trivial effect size

**Mediator Analysis –** none stated

**Study – Reger et al. (2002)**

**Sample –** 31,420 sedentary adults aged 50-65

**Design** – Quasi experimental, 2 arm, 5 measures

**Setting** – community

**PA** **target** – 30 minutes of daily walking

**DV** – Observation and self-report method

**Intervention theory, length, type** – TPB, 8 weeks, community study, target community received paid media and public relations communications for walking, control community no communication

**Outcome** – PA: No significant differences and trivial effect size

**Mediating** **variables** – No differences for attitude or subjective norm. Significant differences in intention and PBC reported but effect sizes were trivial

**Mediator** **Analysis** – none conducted

**Study – Rovniak et al. (2005)**

**Sample –** 50 sedentary women

**Design –** RCT, 2 arm, 11 measures

**Setting –** not specified

**PA target –** walk 3 times/week for 30 minutes at moderate exertion

**DV –** PA: self-report walking logs returned via e-mail and self-report walking quantity questionnaire; Fitness: estimate VO2 max using 1 mile walk test

**Intervention theory, length, type –** SCT, Two 12 week e-mail based intervention with 1 year follow up, 1^st^ intervention group: High theoretical fidelity group incorporated mastery procedures precisely following SCT. 2^nd^ intervention group: low fidelity group incorporated mastery procedures similar to SCT

**Outcomes –** No significant difference between groups at 12 weeks and 1 yr (trivial effect size)

**Mediating Variables –** high fidelity group increased goal setting and positive outcome expectation for walking more that twice as much as low fidelity group. ES=medium. No sig. differences b/w groups in self-efficacy, negative outcome expectation, enjoyment, planning and scheduling, family and friend support change.

**Mediator Analysis –** none stated
