# Supplementary material for: Mediators of physical activity behaviour change among adult non-clinical populations: a review update
Source: Int J Behav Nutr Phys Act. 2010 May 11;7:37. doi: 10.1186/1479-5868-7-37 (PMC2876989; doi:10.1186/1479-5868-7-37)
Supplement: Additional file 5 — Quality of studies using tool developed by Lubans, Foster, and Biddle (2008). This file contains the tool used to assess the quality of each study included in the review. Each study was assessed by a series of questions listed below. BMI: Body Mass Index; RCT: Randomized control trial; PA: physical activity; MI: motivational interviewing; ES: effect size; OR: odds ratio; PAR: physical activity recall. TTM: transtheoretical model; POC: processes of change; SCT: social cognitive theory; IPAQ: International Physical Activity Questionnaire; GLTEQ: Godin Leisure Time Exercise Questionnaire; SDT: self-determination theory; PMT: protection motivation theory; TPB: theory of planned behaviour; PCB: perceived behavioural control; CHD: coronary heart disease. [file 1479-5868-7-37-S5.DOC]

Additional File 5: Quality of studies using tool developed by Lubans, Foster, and Biddle (2008)

|  |  | Questions | | | | | | | | |  |  |  |
| --- | --- | --- | --- | --- | --- | --- | --- | --- | --- | --- | --- | --- | --- |
| Authors | 1 | | 2 | 3 | 4 | 5 | 6 | 7 | 8 | 9 | 10 | 11 | Total Score |
| Ash et al. (2006) |  | |  |  |  |  |  |  |  |  |  |  | 5 |
| Bennett et al. (2008) |  | |  |  |  |  |  |  |  |  |  |  | 4 |
| Bock et al. (2001) |  | |  |  |  |  |  |  |  |  |  |  | 6 |
| Cardinal & Spazianni (2007) |  | |  |  |  |  |  |  |  |  |  |  | 5 |
| Cerin et al. (2006) |  | |  |  |  |  |  |  |  |  |  |  | 4 |
| Cramp & Brawley (2006) |  | |  |  |  |  |  |  |  |  |  |  | 6 |
| Dallow & Anderson (2003) |  | |  |  |  |  |  |  |  |  |  |  | 6 |
| Dinger et al. (2007) |  | |  |  |  |  |  |  |  |  |  |  | 6 |
| Elbel et al. (2003) |  | |  |  |  |  |  |  |  |  |  |  | 4 |
| Farenwahl et al. (2004, 2005) |  | |  |  |  |  |  |  |  |  |  |  | 6 |
| Fortier et al. (2007)  Blanchard et al. (2007) |  | |  |  |  |  |  |  |  |  |  |  | 6 |
| Gallagher et al. (2005) |  | | Yes- for behavioral processes only |  |  |  |  |  |  |  |  |  | 5 |
| Hallam & Petosa (2004) |  | |  |  |  |  |  |  |  |  |  |  | 5 |
| Hurling et al. (2007) |  | |  |  |  |  |  |  |  |  |  |  | 6 |
| Jacobs et al. (2004) |  | |  |  |  |  |  |  |  |  |  |  | 5 |
| Jones et al. (2004) |  | |  |  |  |  |  |  |  |  |  |  | 6 |
| Kinmonth et al. (2008) |  | |  |  |  |  |  |  |  |  |  |  | 7 |
| Kloek et al. (2006) |  | |  |  |  |  |  |  |  |  |  |  | 3 |
| Levy & Cardinal (2004) |  | |  |  |  |  |  |  |  |  |  |  | 6 |
| Lewis et al. (2006) |  | |  |  |  |  |  |  |  |  |  |  | 6 |
| Little et al. (2004) |  | |  |  |  |  |  |  |  |  |  |  | 4 |
| Milne et al. (2002) |  | |  |  |  |  |  |  |  |  |  |  | 6 |
| Napolitano et al. (2008) |  | |  |  |  |  |  |  |  |  |  |  | 6 |
| Parrott et al. (2008) |  | | Yes – for attitude only |  |  |  |  |  |  |  |  |  | 6 |
| Plotnikoff et al. (2005) |  | |  |  |  |  |  |  |  |  |  |  | 6 |
| Reger et al. (2002) |  | |  |  |  |  |  |  |  |  |  |  | 5 |
| Rovniak et al. (2005) |  | |  |  |  |  |  |  |  |  |  |  | 6 |

Questions

1. Did the study include a theoretical framework?
2. Were the methods designed to influence mediator variables?
3. Did the authors report conducting pilot studies to test mediation?
4. Was an objective measure of PA used?
5. Were the measures of PA reliable?
6. Were the mediator measures reliable (e.g., alpha)?
7. Was the study powered to detect mediation?
8. Was the design an RCT?
9. Was baseline PA considered in analyses?
10. Were statistically appropriate/acceptable methods of data analyses used?
11. Did the study ascertain whether changes in the mediator precede changes in the PA outcome?
